# Evolution of the substrate specificity of an RNA ligase ribozyme from phosphorimidazole to triphosphate activation

**DOI:** 10.1073/pnas.2407325121

**Published:** 2024-09-13

**Authors:** Saurja DasGupta, Zoe Weiss, Collin Nisler, Jack W. Szostak

**Affiliations:** ^a^Department of Molecular Biology, Center for Computational and Integrative Biology, Massachusetts General Hospital, Boston, MA 02114; ^b^HHMI, Massachusetts General Hospital, Boston, MA 02114; ^c^Department of Genetics, Harvard Medical School, Boston, MA 02115; ^d^Department of Molecular and Cellular Biology, Harvard University, Cambridge, MA 02138; ^e^HHMI, The University of Chicago, Chicago, IL 60637; ^f^Department of Chemistry, The University of Chicago, Chicago, IL 60637

**Keywords:** in vitro evolution, ribozymes, ligases, RNA world, origins of life

## Abstract

The acquisition of new RNA functions through evolutionary processes was essential for the diversification of RNA-based primordial biology and its subsequent transition to modern biology. However, the mechanisms by which RNAs access new functions remain unclear. Do RNA enzymes need completely new folds to support new but related functions, or is reoptimization of the active site sufficient? What are the roles of neutral and adaptive mutations in evolutionary innovation? Here, we address these questions experimentally by focusing on the evolution of substrate specificity in RNA-catalyzed RNA assembly. We use directed in vitro evolution to show that a ligase ribozyme that uses prebiotically relevant 5′-phosphorimidazole-activated substrates can be evolved to catalyze ligation with substrates that are 5′-activated with the biologically relevant triphosphate group. Interestingly, despite catalyzing a related reaction, the new ribozyme folds into a completely new structure and exhibits promiscuity by catalyzing RNA ligation with both triphosphate and phosphorimidazole-activated substrates. Although distinct in sequence and structure, the parent phosphorimidazolide ligase and the evolved triphosphate ligase ribozymes can be connected by a series of point mutations where the intermediate sequences retain at least some ligase activity. The existence of a quasi-neutral pathway between these distinct ligase ribozymes suggests that neutral drift is sufficient to enable the acquisition of new substrate specificity, thereby providing opportunities for subsequent adaptive optimization. The transition from RNA-catalyzed RNA assembly using phosphorimidazole-activated substrates to triphosphate-activated substrates may have foreshadowed the later evolution of the protein enzymes that use monomeric triphosphates (nucleoside triphosphates, NTPs) for RNA synthesis.

## Significance

The viability of RNA-based primordial life hinges on the ability of RNA to acquire new functions through evolutionary pathways, the details of which are not well understood. Here, we investigate these mechanisms using experimental RNA evolution. We show that RNA enzymes or ribozymes that use prebiotically relevant phosphorimidazolide substrates for RNA ligation can be evolved into ligase ribozymes that use triphosphate substrates. Intriguingly, these ribozymes can change into one another via point mutations without sacrificing enzyme activity, underscoring the ease of functional diversification in RNA-based biology. The emergence of ribozymes that utilize biological triphosphates as substrates for RNA synthesis from more primordial ancestors may have foreshadowed the subsequent emergence of protein-catalyzed RNA synthesis with nucleoside triphosphates.

In an early stage of the evolution of life, referred to as the RNA World, primordial cells are thought to have used RNA to constitute both genomes and enzymes (ribozymes). In the absence of material remains from the RNA World, experimental models are used to understand the various chemical and biochemical processes that would have been important for the origin and evolution of early life. As efficient RNA assembly is a prerequisite for the replication of genomic and catalytic RNAs, there has been considerable focus on modeling RNA ligation and polymerization pathways in the absence and presence of enzymes. Protein enzymes (RNA polymerases) use nucleoside triphosphates (NTPs) as substrates for RNA synthesis. However, the low reactivity of 5′-triphosphorylated monomers/oligomers for nonenzymatic polymerization/ligation suggests that before the emergence of enzymes, RNA building blocks were activated with more reactive groups. 5′-phosphorimidazole-activated monomers and oligomers have been widely used to study nonenzymatic RNA assembly because of their intrinsic reactivity (1–4). Recent discoveries provide strong evidence for the prebiotic relevance of phosphorimidazolides (5–8). To model the crucial transition from nonenzymatic RNA assembly to ribozyme-catalyzed RNA assembly, we previously reported the in vitro selection of ribozymes that use 5′-phosphorimidazolide RNA as substrates for ligation (9).

Although beneficial for both enzyme-free and ribozyme-catalyzed RNA assembly, the enhanced reactivity of phosphorimidazolides makes them susceptible to hydrolysis. Therefore, kinetically more stable 5′-triphosphorylated building blocks would have been better substrates for RNA assembly if they were readily generated on the early Earth and suitable enzymes were available to use them. 5′-triphosphorylated RNA monomers and oligomers can be generated by reactions with abiotically synthesized polyphosphates, such as cyclic-trimetaphosphate (10–12) and ribozymes that accelerate this triphosphorylation reaction have been reported (13, 14). Additionally, triphosphate substrates may be produced by the reaction of phosphorimidazolides with pyrophosphate, the latter abundantly generated in mineral films in alkaline hydrothermal environments (15–17). As the uptake of trimetaphosphate or triphosphorylated RNA oligonucleotide substrates by primitive cells seems unlikely, given that even NTPs show low permeability to fatty acid vesicles (18), triphosphorylated substrates could plausibly be generated by the intracellular conversion of phosphorimidazolides to triphosphates through reactions with encapsulated pyrophosphate.

The availability of 5′-triphosphorylated building blocks would impart a selective advantage to ribozymes that used such substrates. Several ribozymes that use 5′-triphosphorylated substrates have been artificially evolved (3, 19–27). But how, in the RNA World, might ribozymes that used phosphorimidazolides have acquired the ability to use triphosphates? The chemistry of RNA assembly with these two substrates differs only in the identity of the leaving group (imidazole vs. pyrophosphate). Would a minor reorganization of the active site allow ligases to switch from phosphorimidazolides to triphosphates, or is a completely new catalytic fold required? Results from RNA evolution experiments where new functions were evolved from existing ones indicate that sequences often need to diverge greatly to access new functions (28–33). This observation is intriguing as RNA fitness landscapes are considered to be rugged and composed of isolated high-activity peaks separated by low-activity valleys (34–38). As evolutionary adaptation is conceptualized as the climbing of fitness peaks, adaptation across rugged landscapes is expected to be difficult. However, functional RNAs can be robust to mutational perturbation, resulting in the existence of mutational pathways composed of functional sequence variants (“neutral pathways”) between fitness peaks (39, 40). Therefore, new RNA functions may be accessed via neutral or quasi-neutral drifts across the fitness landscape followed by adaptive optimization (i.e., peak climbing). Although it has been suggested that neutral networks pervade the RNA sequence space and connect distinct phenotypes (41–43), only a handful of examples have been reported (37, 43, 44). Understanding the role of neutral pathways in the evolution of new functions in RNA is crucial for explaining the evolutionary diversification of ribozymes and, consequently, the emergence of RNA-based biology.

Here, we report the in vitro evolution of a new ligase that uses 5′-triphosphorylated substrates (“PPP-ligase”) starting from a previously characterized ligase ribozyme that uses 5′-phosphoro-2-aminoimidazolide activated RNA oligonucleotides as substrates (“AIP-ligase”). The PPP-ligase ribozyme differs from the parent AIP-ligase by 28 mutations and consequently adopts a new structure. The new PPP-ligase, in addition to catalyzing the ligation of triphosphorylated substrates, also catalyzes the ligation of phosphorimidazolide substrates, making it catalytically promiscuous. Despite being separated by 28 mutations, the parent AIP-ligase and the newly evolved PPP-ligase are connected by a quasi-neutral pathway where each point mutant catalyzes ligation with either phosphorimidazolide or both phosphorimidazolide and triphosphate substrates. Promiscuous ribozymes with the capacity to catalyze RNA assembly using both prebiotically relevant phosphorimidazolides and biologically relevant triphosphates could have led to the evolution of enzymatic pathways for the synthesis of triphosphate substrates. This may have foreshadowed the later emergence of protein polymerases that use the same triphosphate substrate activation that is now universal in biology.

## Results

### Isolation of New Ligase Ribozymes through Directed Evolution.

**Fig. 1.**
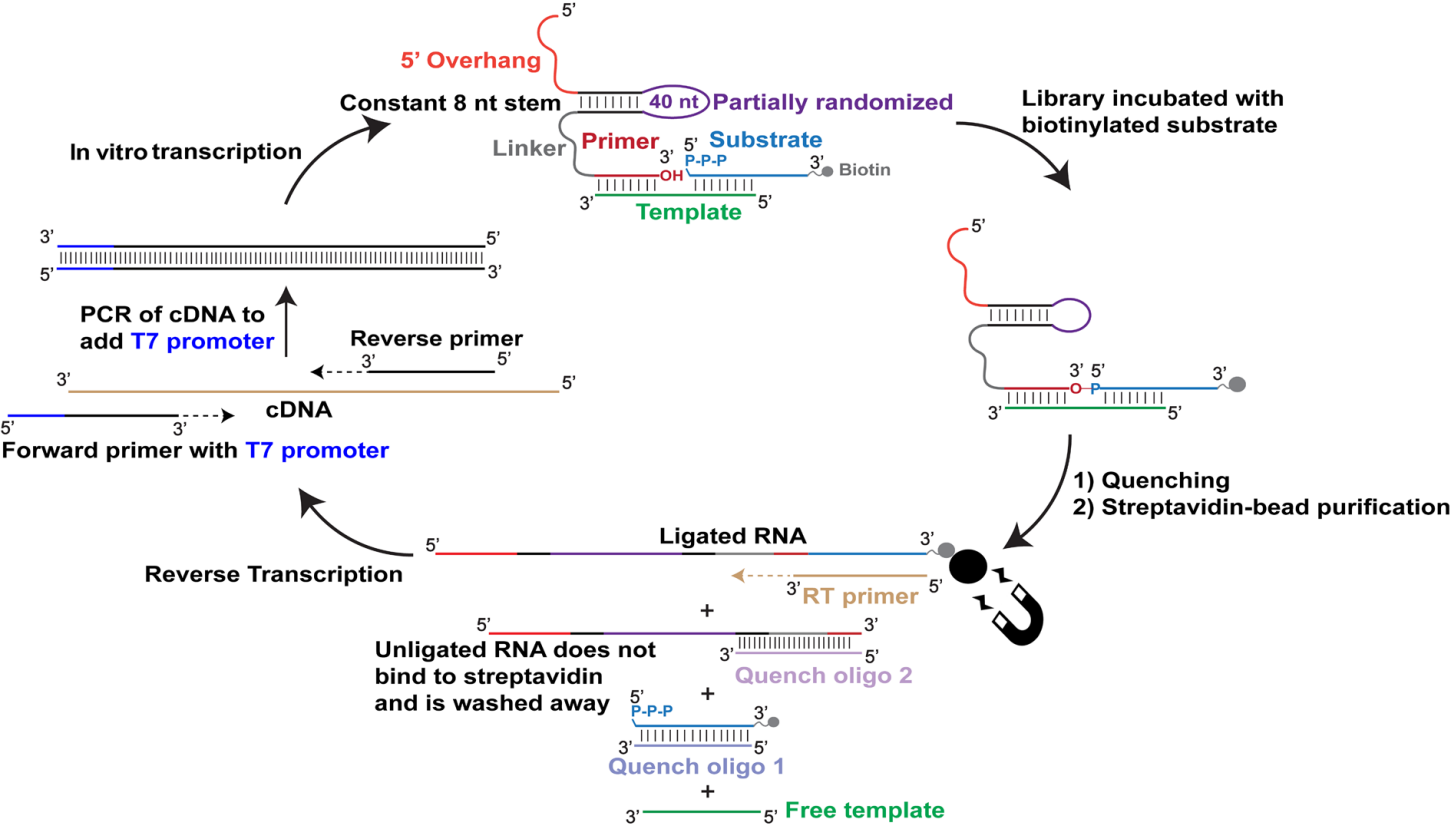
Selection strategy to isolate ribozymes that catalyze the ligation of 5′-triphosphorylated RNA. A partially randomized library derived from an existing AIP-ligase was incubated with an RNA template and a 5′-triphosphorylated RNA substrate that was biotinylated at its 3′ end. Reactions were quenched by adding EDTA. Excess DNA oligos complementary to the substrate and the 3′ ends of unligated library sequences were added to disrupt the ternary complex formed by the library, template, and substrate sequences, thus preventing the retention of inactive sequences on beads due to noncovalent association with the biotinylated substrate. Sequences that catalyzed ligation were captured on streptavidin-coated magnetic beads and reverse transcribed using a primer that is complementary to the substrate, thus forcing the selection of the desired ligases. The resulting cDNA was converted to dsDNA and amplified by PCR, which also added a T7 promoter sequence to the dsDNA. The dsDNA was in vitro transcribed to generate the RNA library for the next round of selection.

**Fig. 2.**
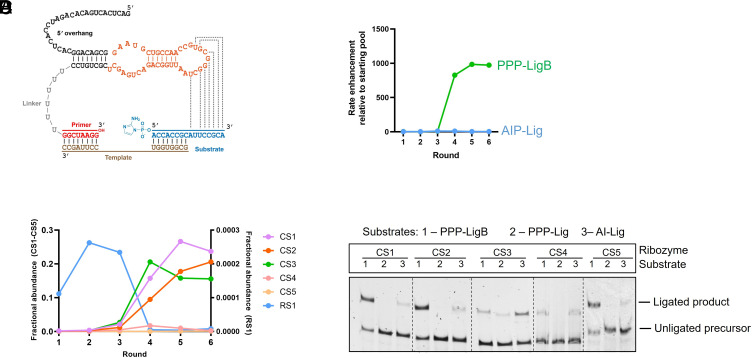
Enrichment of catalytically active sequences during selection and their biochemical validation. (*A*) Secondary structure of the parent AIP-ligase ribozyme that was partially mutagenized to generate the selection library. The library region is highlighted in orange. Dashed lines indicate putative base-pairing interactions between the ribozyme and the substrate. (*B*) Significant enrichment in ligase activity with PPP-LigB (~1,000-fold) was observed after round 4, which was maintained through round 6. (*C*) Certain sequences became predominant after round 4, which coincided with the abrupt fall in the relative abundance of the parent AIP-ligase, RS1. Fractional abundances of CS1 to CS5 and RS1 are shown in the left and right y-axes, respectively. (*D*) Ligase activities of the five most abundant sequences with 1) 5′-triphosphorylated and 3′ biotinylated substrate (PPP-LigB), 2) 5′-triphosphorylated substrate (PPP-Lig), and 3) 5′-phosphorimidazolide substrate (AIP-Lig). Only CS3 is able to ligate PPP-Lig and AI-Lig. Ligation reactions contained 1 µM ribozyme, 1.2 µM RNA template, and 2 µM RNA substrate in 100 mM Tris-HCl pH 8.0, 300 mM NaCl, and either 10 mM MgCl_2_ (for ligation with AIP-Lig) or 100 mM MgCl_2_ (for ligation with PPP-Lig/PPP-LigB).

In order to select new ligase ribozymes that use 5′-triphosphorylated substrates, we prepared a sequence library derived from a previously characterized AIP-ligase ribozyme (hereafter, RS1) that comprised a 40 nt catalytic domain flanked by an 8 base-pair stem (Figs. 1 and 2*A*). The catalytic domain was partially randomized at 21% per position (i.e., 7% each of the three non-RS1 nucleotides) to generate the selection library (wild type RS1 = 0.008%). The library was connected to an 8 nt primer sequence at its 3′ end via a U_6_ linker. The constant 5′ and 3′ regions contained primer binding sites for PCR amplification. We started with 3 nmol of the RNA library transcribed from 1.2 nmol dsDNA (estimated complexity of ~10^15^ sequences). The library was challenged with a 5′-triphosphorylated RNA oligonucleotide substrate that was biotinylated at its 3′ end (“PPP-LigB”). An external RNA template was supplied to bring the primer (i.e., 3′-end of the library RNAs) and the substrate into close proximity to facilitate the targeted reaction between the primer 3′-OH and the substrate 5′-α-phosphate (Fig. 1). Active library sequences were affinity-purified using streptavidin-coated magnetic beads by virtue of their covalent attachment to the biotin-tagged substrate. The captured sequences were selectively amplified by RT-PCR using appropriate primers (*SI Appendix*, Table S6) and in vitro transcribed to generate an enriched library for the next round of selection (Fig. 1). Selection stringency was increased by reducing the reaction time and/or lowering the Mg^2+^ concentration in each round to isolate the most active ribozymes (*SI Appendix*, Table S1).

We assayed ligase activities of the output library from each round and observed significant activity after round 4 (Fig. 2*B*). While the starting “round 0” library demonstrated low but detectable ligation with PPP-LigB (*k*_obs_ = 0.00094 ± 0.0001 h^−1^), no ligation was detected after 3 d with a completely randomized library. After round 6, the library was found to ligate the selection substrate, PPP-LigB, three orders of magnitude faster than the starting library. In contrast, both the round 6 library and the starting library exhibited comparable ligase activities toward an identical RNA sequence with a 5′-phosphoro-2-aminoimidazolide group (“AIP-Lig”) (Fig. 2*B*).

High-throughput sequencing of the output from each round of selection revealed significant enrichment of specific sequences after round 4, with the three most abundant sequences comprising ~60% of the total reads in round 6 (Fig. 2*C* and Table 1). Early rounds of selection showed a small increase in the representation of the parental AIP-ligase, RS1 (Fig. 2*C*), from an expected abundance of ~0.01% in the initial doped library to 0.025% after round 2 and round 3, after which the parental sequence exhibits a sharp decline in abundance. Further rounds of selection led to a continuing decrease in the sequence diversity of the sequence library (*SI Appendix*, Table S1). The decline in both the abundance of RS1 and the overall sequence diversity, together with the emergence of new dominant sequences, suggested the isolation of new ligase ribozymes.

**Table 1.** The five most abundant sequences identified by high-throughput sequencing

	Library sequence (40 nt, 5′→3′)	% abundance of peak sequence within its cluster	% abundance of peak sequence (overall)	% abundance of total cluster (overall)
CS1	GACAGCCGAGAAAUGAGUGGCCUAAAUGGGAGAAUGAGCU	65.41	23.68	36.21
CS2	GACUGCGCGUAUGAGUGGCGGCUAAAGAGGAGAAUGAGCG	81.61	20.59	25.23
**CS3**	**ACGGGUGGGUAAUCUAGUGUCCGCGGAAUAGAACGAAACA**	**49.97**	**15.55**	**31.12**
CS4	GGAUGGUGCGAACUGAGUGGGCUAAUUAGGAGAAUGAGCG	30.93	0.23	0.73
CS5	GGAGGGUGACAUCGUUGAGAGAGAAUGGGGAUAUUGAACU	13.40	0.12	0.88

Sequences CS1 to CS5 represent the most abundant sequences in their respective clusters (referred to as peak sequences). While the peak sequences in clusters CS1 to CS5 occupied ~60% of the round 6 population, the clusters as a whole accounted for ~90% of all sequences. The PPP-ligase, CS3, which occupied ~15% of the round 6 population, is shown in boldface. The 40 nt library sequences shown here constitute the partially randomized region highlighted in orange in Fig. 2*A*.

### A Single Ribozyme Class Catalyzes the Ligation of 5′-Triphosphorylated RNA.

Sequences isolated from round 6 were sorted into distinct clusters of closely related sequences with little sequence overlap between clusters. Sequences in the three most abundant clusters comprised 92% of all sequence reads, and the dominant sequences (hereafter, peak sequences) of each cluster represented 50 to 80% of all reads within their clusters (Table 1). To capture the diversity of the selected ligases, all peak sequences that occupied >0.1% of the total sequence reads were tested for their ability to ligate to the selection substrate, PPP-LigB, in addition to a triphosphorylated substrate that lacked a biotin tag (PPP-Lig) and the phosphorimidazolide substrate, AIP-Lig. Peak sequences from CS1-CS5, representing the five most abundant ligase clusters, satisfied this threshold. While CS3 catalyzed ligation with all three substrates, CS1, CS2, CS4, and CS5 catalyzed ligation with only PPP-LigB (Fig. 2*D*). The failure to ligate PPP-Lig suggests that CS1, CS2, CS4, and CS5 do not catalyze the desired reaction, but survived selection by using alternative ligation mechanism(s) (a detailed analysis will be reported elsewhere). On the other hand, CS3’s ability to ligate both phosphorimidazolide and triphosphate substrates indicated that we had isolated a promiscuous ligase.

### Ligation Requires a Template and Generates a 3′-5′ Phosphodiester Linkage.

**Fig. 3.**
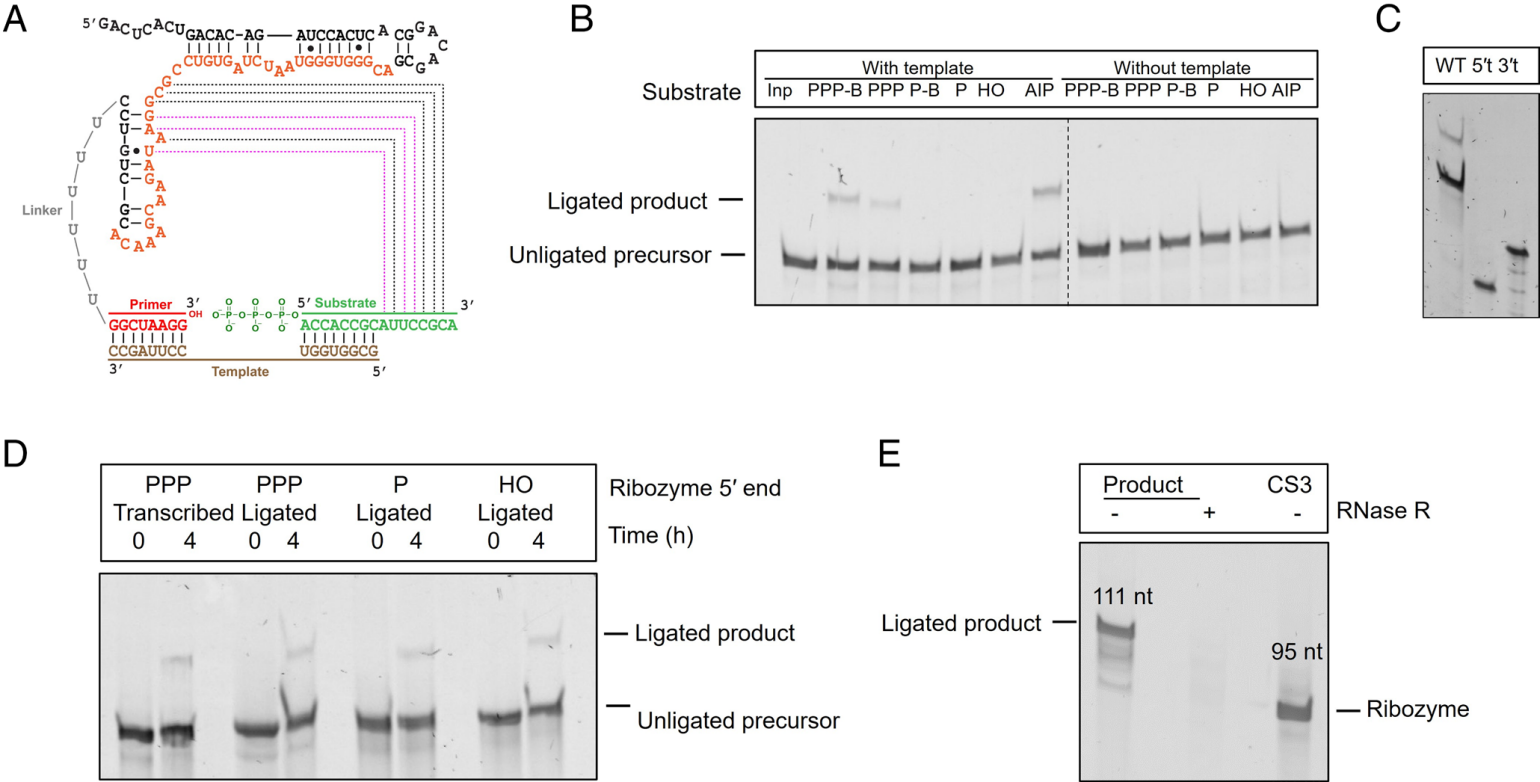
CS3-catalyzed templated ligation to triphosphate substrates involves the 3′-hydroxyl group of the primer and the 5′-triphosphate group of the substrate. (*A*) SHAPE-derived secondary structure of CS3 in complex with the template and substrate RNA sequences (*SI Appendix*, Fig. S5 for SHAPE data). The region mutagenized in the library is highlighted in orange. Dashed lines indicate putative base-pairing interactions between the ribozyme and the substrate (see below). Black and magenta lines indicate complementarity between the substrate 3′ end and the unpaired and paired residues of the ribozyme, respectively. (*B*) CS3-catalyzed RNA ligation requires a template and 5′ substrate activation, either in the form of triphosphate or phosphorimidazole. PPP-B and P-B indicate PPP-LigB and PLigB, respectively. (*C*) Truncation of CS3 by deleting the first 25 nt from the 5′ end or the last 14 nucleotides from the 3′ end results in the loss of activity. (*D*) CS3 tolerates changes to its 5′ chemistry, indicating that its 5′ end does not participate in ligation. CS3 variants with 5′P/OH were generated by splinted RNA ligation (*Materials and Methods*). 5′PPP-CS3 constructs generated through splinted ligation and in vitro transcription were used as positive controls. (*E*) The 111 nt product of ligation between CS3 and PPP-Lig is completely degraded by RNase R, which suggests that it contains a 3′-5′ linkage between the ribozyme and substrate. The product lanes contain purified ligated product, incubated with or without RNase R. Ligation reactions contained 1 µM ribozyme, 1.2 µM RNA template (in templated reactions), and 2 µM RNA substrate in 100 mM Tris-HCl pH 8.0, 300 mM NaCl, and either 10 mM MgCl_2_ (for ligation with AIP-Lig) or 100 mM MgCl_2_ (for ligation with PPP-Lig/PPP-LigB/P-LigB, P-Lig, OH-Lig).

To confirm that CS3 catalyzes the desired reaction, we first tested substrates with different 5′ chemistries, with or without a template (Fig. 3 *A* and *B*). Ligation was eliminated in the absence of the template. Substrates with unactivated 5′-monophosphates, with or without 3′ biotin (P-B or P) failed to undergo ligation, while 5′-triphosphate substrates were ligated by CS3. CS3 also ligated to a substrate with 5′-phosphorimidazole activation (AIP). Next, we tested CS3 truncation constructs in which either the first 25 nt, consisting of the PCR primer binding sequence (5′t) or the last 14 nt, including the 3′ primer (3′t), were deleted. Both truncations abolished ligation (Fig. 3*C*). As the 3′ truncation involves the deletion of a presumably unstructured region (U_6_ linker + 8 nt primer), loss of activity due to ribozyme misfolding is unlikely. This implicated the 3′ primer sequence in CS3-catalyzed ligation. The deleted 5′ sequence in 5′t, on the other hand, base-pairs extensively with the central 40 nt region corresponding to the mutagenized segment (Fig. 3*A*) (*SI Appendix*, Fig. S5 for secondary structure determination by SHAPE probing), explaining the loss of activity upon 5′ truncation. Previous selections targeting 3′-5′ ligation have resulted in the isolation of ribozymes that catalyze ligation between the ribozyme 5′-triphosphate and the substrate 5′-phosphorimidazole group (45). To test this possibility, we examined the activities of CS3 variants containing either a 5′-monophosphate (5′P) or a 5′-hydroxyl (5′OH) moiety. As expected, both CS3 variants exhibited PPP-ligation, consistent with ligation at the 3′ end of the ribozyme (Fig. 3*D*).

With strong support for the participation of the ribozyme 3′ end and substrate 5′ end, we investigated the importance of base-pairing at the ligation junction by mutating the relevant template nucleotides. A C → G mutation that disrupts a base-pair between the 3′ terminal nucleotide of the primer and the template preserved ligation, but a U → A mutation that disrupts an A-U base-pair between the 5′ terminal nucleotide of the substrate and the template, abrogated ligation (*SI Appendix*, Fig. S1). The parent AIP-ligase, RS1, generates a 3′-5′ linkage, which is the canonical regiochemistry of RNA backbones in biology. To characterize the linkage created as a result of CS3-catalyzed ligation with PPP-Lig, we purified the ligated product and incubated it with RNase R, a 3′ → 5′ exonuclease. We observed complete degradation of the ligated product indicating the presence of a natural 3′-5′ linkage as opposed to a 2′-5′ linkage, which would result in exonuclease stalling at the linkage (Fig. 3*E*). Collectively, these results confirmed that CS3-catalyzes the desired reaction, i.e., templated ligation of 5′-triphosphorylated RNA generating a 3′-5′ phosphodiester linkage between the ribozyme and the substrate.

### Comparison with Other Ribozyme-Catalyzed Ligation Reactions.

**Fig. 4.**
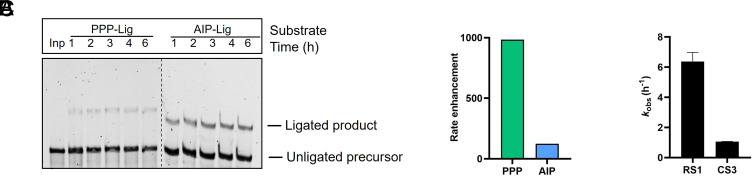
CS3 catalyzes the ligation of both triphosphate and phosphorimidazolide RNA substrates. (*A*) Representative time course for CS3-catalyzed ligation of PPP- and AIP-substrates. (*B*) CS3 accelerates PPP-ligation by ~1,000-fold and AIP-ligation by ~100-fold relative to their corresponding background reactions. (*C*) CS3 is ~six-fold slower than the parent AIP-ligase, RS1 for AIP-ligation. Ligation reactions contained 1 µM ribozyme, 1.2 µM RNA template, and 2 µM RNA substrate (AIP-Lig or PPP-Lig) in 100 mM Tris-HCl pH 8.0, 300 mM NaCl, and either 10 mM MgCl_2_ (AIP-Lig) or 100 mM MgCl_2_ (PPP-Lig).

Template-directed nonenzymatic RNA ligation has been explored for the last 50 y as a model for prebiotic RNA assembly (46, 47). Imidazoles are superior leaving groups to pyrophosphates, therefore, templated phosphorimidazolide ligation, unlike ligation with triphosphorylated RNA, is detectable even in the absence of enzymes. For example, at pH 8 and 100 mM Mg^2+^, *k*_obs_ for template-directed nonenzymatic ligation with triphosphorylated RNA is ~1.3 × 10^−5^ h^−1^, while the corresponding *k*_obs_ with a 2AI-activated RNA is 2.4 × 10^−2^ h^−1^_,_ approximately three orders of magnitude faster (9, 47). While previously isolated AIP-ligases exhibit rate enhancements of 170 to 740-fold (at 10 mM Mg^2+^) (9), the fastest PPP-ligases accelerate ligation by 10^6^-fold (at 100 mM Mg^2+^) (19, 21, 48, 49). CS3, although specifically selected for PPP-ligation, also catalyzes AIP-ligation (Fig. 4*A*). CS3 accelerates PPP-ligation by ~10^3^-fold at 100 mM Mg^2+^, and AIP-ligation by ~100-fold at 10 mM Mg^2+^ (Fig. 4*B*), making it slower than most of the previously reported PPP-ligases and AIP-ligases (Fig. 4*C*).

Ribozymes often increase reaction rates by tightly binding and positioning catalytic Mg^2+^ ion(s) within the active site (9, 19, 48, 50). The first ligase ribozyme reported to use triphosphorylated RNA substrates (“class I” ligase) uses Mg^2+^ for neutralizing the negative charge on the pyrophosphate group and activating the 3′-O nucleophile (51), strategies also used by protein-based polymerases (52–56). A Mg^2+^-titration curve for CS3-catalyzed PPP-ligation revealed a sharp increase in *k*_obs_ between 5 mM and 25 mM followed by a plateau of the rate constant with increasing Mg^2+^ concentration (*SI Appendix*, Fig. S2*A*). The binding curve yielded a [Mg^2+^]_1/2_ value of ~14 mM and is consistent with 3 bound Mg^2+^ ions. Since deprotonation of the 3′ OH is an important step in ligation, we measured *k*_obs_ at pH values between 6 and 10 (*SI Appendix*, Fig. S2*B*). Ligation rates increased between pH 6 and pH 9 and then fell, as in the corresponding uncatalyzed reaction (47). The “log *k*_obs_ vs. pH” curve is linear between pH 6 and pH 9 in both CS3-catalyzed and uncatalyzed PPP-ligation. While a slope of 1, as observed for the uncatalyzed reaction likely indicates 3′ OH deprotonation in the rate-determining step, the slope of 0.45 for the CS3-catalyzed ligation may reflect a change in mechanism. Similar deviations were observed for the parent AIP-ligase, RS1 (9), a DNAzyme that ligates imidazole-activated RNA (57), and for variants of the class I ligase (58).

The catalytic promiscuity of CS3, as evidenced by its ability to catalyze ligation with both PPP-Lig and AIP-Lig, was surprising as its parent, RS1, is specific to 5′-monophosphorylated RNA oligonucleotides activated with 2-aminoimidazole and does not ligate triphosphorylated RNA (9). The specificity of RS1 is underscored by its lack of reactivity toward a substrate activated with 2-methylimidazole (MeIP), which suggests that RS1 may interact with the amino group of 2-aminoimidazole. In contrast, CS3 ligates MeIP-Lig, albeit less efficiently than AIP-Lig (*SI Appendix*, Fig. S3). This observation is consistent with CS3 having a flexible or open active site that accommodates diverse leaving groups such as pyrophosphate, 2-aminoimidazole, and 2-methylimidazole.

### Acquisition of a New Function Is Accompanied by the Emergence of a New Structure.

The SHAPE-derived secondary structure of the catalytic domain of the parent AIP-ligase, RS1, consists of two stems interrupted by an internal loop with the outer stem closed by a hairpin loop (*SI Appendix*, Fig. S4*A*). The inner stem, which was part of the constant region in the library, is flanked on its 5′ side by a 25 nt sequence that was designed as a PCR primer binding site and a U_6_ linker connected to the 8 nt primer sequence on its 3′ side (9). SHAPE probing of CS3 revealed a new secondary structure, which is composed of two stems interrupted by internal bulges and terminating in stem-loops (*SI Appendix*, Fig. S5). These stems are connected by a stretch of unpaired nucleotides, which could potentially act as a hinge between these two stem regions. The variable region (shown in orange in Fig. 3*A*) extensively base pairs with the fixed 5′ primer binding region and the nucleotides that formed the 3′ strand of the inner stem in the RS1 structure (Figs. 2*A* and 3*A*). The loss of activity upon 5′ truncation (Fig. 3*C*) and a high degree of nucleotide conservation in the variable region (*SI Appendix*, Fig. S6) are both consistent with this secondary structure. A stretch of 7 nucleotides (5′-GCGGAAU-3′) connecting the two stem regions is perfectly complementary to part of the substrate that remains unpaired upon template binding, referred to here as the 3′ overhang (5′-AUUCCGC-3′) (*SI Appendix*, Fig. S4*B*). While some of these nucleotides are free to pair with the substrate (complementarity is indicated by black dashed lines), nucleotides 3 to 5 (5′-GGA-3′) may be base paired within the ribozyme secondary structure (complementarity with the substrate is indicated by magenta dashed lines). Disrupting the putative base-pairing between CS3 and the substrate by sequestering the substrate 3′ overhang with longer templates eliminated ligation (*SI Appendix*, Fig. S4 *B* and *D*). Similar results were obtained with RS1, where nucleotides in its stem-loop [nucleotides (16–21, 23)] were complementary with the substrate 3′ overhang (*SI Appendix*, Fig. S4 *A* and *C*). These results suggest that both CS3 and RS1 use base-pairing with the substrate to assemble the enzyme–substrate complex. Despite having distinct sequences and structures, the two ligases have converged on a similar solution to substrate binding.

The structural differences between RS1 and CS3 were further highlighted by a combination of SAXS analysis and molecular dynamics simulation (*SI Appendix*, Fig. S7). First, we used SAXS to generate low-resolution molecular envelopes for RS1 and CS3. Next, we used Rosetta’s FARFAR2 (59) method to generate molecular models for RS1 and CS3 with the corresponding SHAPE-derived secondary structures included as constraints. These computational models were used as starting points for all-atom molecular dynamics simulations. Computed molecular models of RS1 and CS3 were fitted to experimental SAXS envelopes (*SI Appendix*, Fig. S7 *A*–*F*). While the best-computed model of RS1 agreed with the SAXS envelope, the best fit of CS3 to the SAXS data required a two-state model (*SI Appendix*, Fig. S7 *C* and *F*). The structural ensembles qualitatively suggest a more dynamic structure for CS3, which agrees with the two-state fit to the SAXS data. A higher degree of flexibility in the CS3 structure is also indicated by a greater RMSD value of the CS3 structure during the simulation with respect to the starting conformation (*SI Appendix*, Fig. S7 *G* and *H*). The inclusion of the substrate/template duplex in the molecular models of RS1 and CS3 revealed a similar dynamic relationship between the substrate/template duplex and the respective ribozyme. RS1 and CS3 each adopt similar conformations in the presence and absence of the substrate/template duplex (*SI Appendix*, Fig. S8*A*), providing further validation for the structures used in the SAXS analysis (*SI Appendix*, Fig. S7). Additionally, the structural fluctuations observed in RS1 and CS3 during simulations are qualitatively similar both in the absence and presence of the substrate/template duplex (*SI Appendix*, Figs. S7*H* and S8*B*). However, inclusion of the substrate/template duplex reveals a difference in the dynamics of the hinge region, which is formed by an unstructured U_6_ linker in RS1 and by stem P2 in CS3. Fluctuations in the hinge region are likely responsible for bringing the ligation junction closer to the ribozyme catalytic core. Next, we included interactions between the ribozyme and the substrate within the above “substrate-unbound” CS3 model to generate a “substrate-bound” CS3 model. We found that the dynamic nature of the hinge region allows the “substrate-unbound” structure to approach the ligation-competent “substrate-bound” structure. Finally, in addition to forming the structural hinge, the P2 stem in the “substrate-bound” CS3 structure interacts physically with the ligation site and coordinates metal ions (*SI Appendix*, Fig. S8*D*). These results point to the importance of the hinge sequence in CS3 function.

### Population Dynamics of the Selected Ligases.

**Fig. 5.**
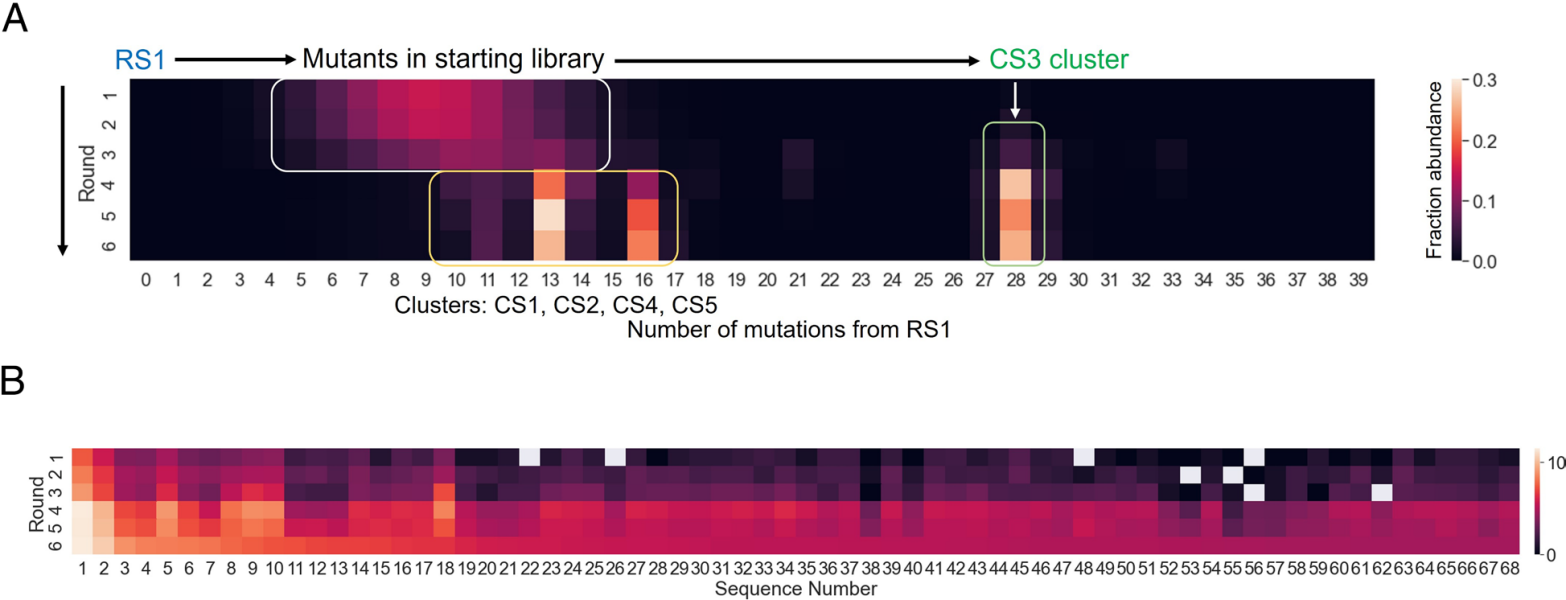
RNA population dynamics during in vitro evolution. (*A*) The selected sequence population moved farther from the parent sequence, RS1, along the selection trajectory forming distinct clusters. While ligases CS1, CS2, CS4, and CS5 and their close variants were 10 to 16 mutations away from RS1, the PPP-ligase, CS3, was 28 mutations from RS1. The colors of the heat map indicate fractional abundance in the entire sequenced pool (see heat map legend). Sequence variants of RS1 present in rounds 1 to 3 are highlighted by a white box, isolated sequences CS1, CS2, CS4, and CS5 that catalyze ligation using alternate pathways are highlighted by a yellow box, and triphosphate ligase CS3 is highlighted by a green box. Only sequences that have >100 reads are shown. (*B*) Sequence dynamics in the CS3 cluster. The peak sequence, CS3 (Sequence 1), was already selected in round 1 and became more abundant during selection. Certain sequences like Sequences 53, 55, 56, and 62 fell below the read threshold but reemerged in later rounds. *SI Appendix*, Table S2 for information about the sequences and their relative abundance. Colors indicate the log of read counts within the CS3 cluster (see heat map legend). Only sequences that have >100 reads are shown.

The parent AIP-ligase ribozyme, RS1, underwent drastic changes to its catalytic scaffold to adapt to PPP-ligation (in CS3). This is a direct consequence of the large distance of 28 mutations between RS1 and CS3, which represents 70% sequence change within the 40 nt variable region, and an overall change of ~30% (28 nt/95 nt). This large mutational distance was unexpected, considering that the level of mutagenesis in the starting library was only 21%. The population of RS1 variants moved farther from RS1 along the selection trajectory, as visualized by high-throughput sequencing (Fig. 5*A*). Sequences 8 to 11 mutations from RS1, were the most abundant after round 1 but the maximum of this distribution shifted to variants with 10 to 13 mutations after round 3. After round 4, concurrent with detectable ligase activity, the selected RNA population became dominated by three distinct subpopulations that were 13, 16, and 28 mutations from RS1. Sequences 13 and 16 mutations from RS1 represent unrelated ligase clusters CS1 and CS2 + CS5, respectively. Sequences 28 mutations from RS1 represent the PPP-ligase cluster, CS3. Sequence variants of RS1 present in rounds 1-3 are highlighted by a white box, isolated sequences CS1, CS2, CS4, and CS5 that catalyze ligation using alternate pathways are highlighted by a yellow box, and triphosphate ligase CS3 is highlighted by a green box. Although CS3 and RS1 catalyze similar ligation reactions, CS3 is mutationally farther from RS1 than the unrelated ligases. The isolation of variants that differ significantly from the parent sequence to access related functions has been observed consistently during in vitro evolution experiments (28–33).

To study the population dynamics of sequences within the CS3 cluster, we plotted the fractional abundance of all CS3 variants with >100 reads for each round (Fig. 5*B* and *SI Appendix*, Table S2). This allowed us to track how the abundance of each sequence in the CS3 cluster fluctuated across rounds. Most sequences become more abundant during selection, with a sudden enrichment after round 4. While the peak sequence, CS3 (sequence 1 in Fig. 5*B*), remained dominant in the cluster throughout, ultimately populating >50% of the cluster, certain sequences such as 22, 26, and 48 became gradually more abundant after emerging in round 2 (i.e., with >100 reads). On the other hand, the abundance of sequences 53, 55, 56, and 62 fell below the read threshold but then reemerged later during the selection. Tracking the relative abundances of PPP-ligase variants illuminates how these ribozyme sequences respond to changing selection pressures.

### A Quasi-Neutral Pathway Facilitates Evolutionary Innovation.

**Fig. 6.**
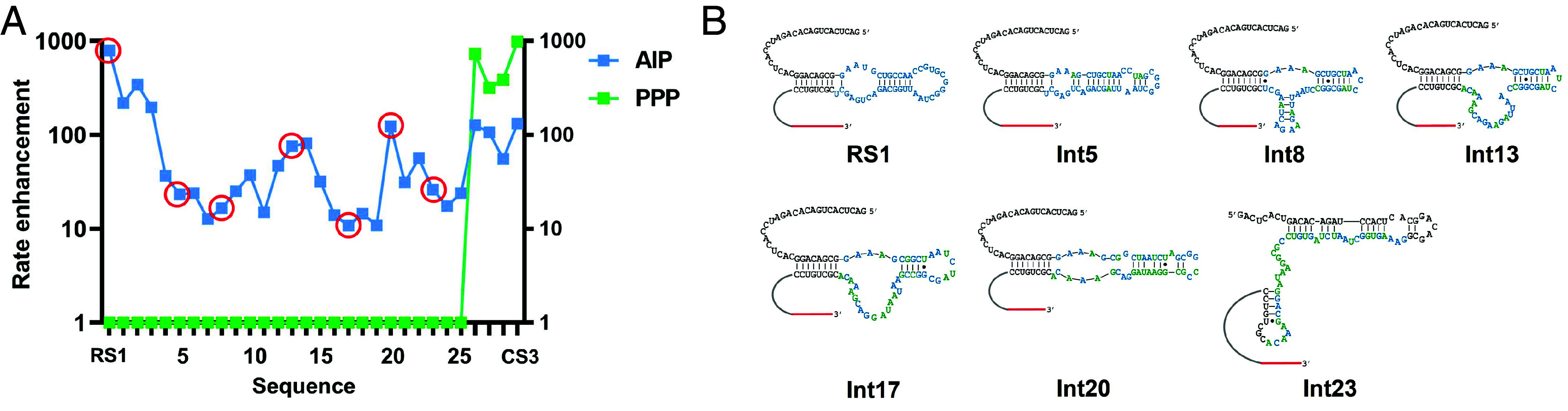
A quasi-neutral mutational pathway connecting ribozyme-catalyzed phosphorimidazolide and triphosphate RNA ligation. (*A*) Mutational path between RS1 and CS3 showing intermediates that differ by single-step mutations. AIP-ligation and PPP-ligation are depicted in blue and green, respectively. Each sequence, represented by a data point, is separated from its immediate neighbors by a single mutation (i.e., Hamming distance of one). An accumulation of point mutations to RS1 causes a fall in AIP-ligase activity, which fluctuates until PPP-ligase activity emerges in Int26 (INT4 in *SI Appendix*, Table S3), which is also accompanied by an increase in AIP-ligase activity. This represents the emergence of a new stable but promiscuous catalytic fold. Int1-4 are predicted to adopt an RS1-like structure, Int23-28 are predicted to adopt a CS3-like structure, and Int5-22 are predicted to fold into structures that can be grouped into five distinct structural folds. (*B*) The seven distinct structural folds in the quasi-neutral pathways from RS1 to CS3. The intermediates shown here are the sequences where these structural folds first emerge. These sequences are highlighted in red circles in (*A*). The computationally predicted secondary structures of all the sequences in (*A*) can be found in *SI Appendix*, Fig. S9. Sequences used in this analysis are listed in *SI Appendix*, Table S4. Ligation reactions in (*A*) contained 1 µM ribozyme, 1.2 µM RNA template, and 2 µM RNA substrate (AIP-Lig or PPP-Lig) in 100 mM Tris-HCl pH 8.0, 300 mM NaCl, and either 10 mM MgCl_2_ (AIP-Lig) or 100 mM MgCl_2_ (PPP-Lig).

The large mutational distance between the AIP-ligase, RS1, and the PPP-ligase, CS3, raises questions about the feasibility of adaptive mutational walks along the ligase fitness landscape. It has been proposed that the RNA sequence space is permeated by neutral networks in which each mutant intermediate is one mutation from its immediate neighbors and retains fitness (41, 43, 60). The existence of neutral pathways connecting distinct RNAs drastically reduces the need for mutations that significantly increase fitness (adaptative mutations). While our results show that a PPP-ligase can be evolved from an AIP-ligase in a single jump following mutagenesis, we wondered whether these ligases are connected by neutral pathways, where each single-step mutant intermediate retains at least some ligase function. To identify these neutral intermediates, we computationally generated all possible single mutants of CS3 and ran Dijkstra’s shortest path finding algorithm from RS1 to these single mutants, using only the sequences present in the sequencing data obtained from rounds 1-6. The algorithm was designed to identify the path that terminated in a sequence that most closely resembles CS3. Unfortunately, the path terminated in a sequence that was only 2 mutations from RS1 (INT3 in *SI Appendix*, Table S3), suggesting the absence of intermediates between RS1 and CS3 in the sequencing data. Next, we repeated this step by running our algorithm from CS3 to all single mutants of INT3. This search terminated at INT4 (*SI Appendix*, Table S3), which is 24 mutations from INT3 and only 3 mutations from CS3. Collectively, this computational approach revealed six intermediate sequences present in the sequencing data—three closely resembling RS1 (INT1-INT3) and three closely resembling CS3 (INT4-INT6), with a 24-mutation gap between INT3 and INT4 (*SI Appendix*, Table S3). This gap does not necessarily indicate the absence of a neutral path. Active sequence variants of AIP-ligase RS1 that were present in the starting library were removed during selection as our experiment was designed to select for PPP-ligase activity. As a result, the selected populations do not contain mutants that are closely related to RS1. Moreover, our experiment does not sample the entire sequence space between RS1 and CS3. Therefore, we took an experimental trial and error approach to fill this gap by designing and assaying the AIP and PPP ligase activities of mutant sequences that complete a single-step mutational pathway between INT3 and INT4. Only mutant sequences with rate enhancements that were >10% of that exhibited by the target sequence, CS3 were considered as “quasi-neutral” intermediates and included in our analysis (Fig. 6*A* and *SI Appendix*, Table S4). As CS3-catalyzed AIP ligation and PPP ligation are ~100- and ~1,000-fold faster than their background reactions, respectively, intermediates had to show rate enhancements of 10-fold for AIP-ligation or 100-fold for PPP-ligation to be considered quasi-neutral.

INT4 (mutant 26 in Fig. 6*A*), which represents the mutational jump from the AIP-ligase phenotype to the PPP-ligase phenotype, catalyzed both AIP and PPP-ligation as expected by its sequence similarity to CS3. The two intermediates between INT4 and CS3 were also active for both functions. We made sequential mutations to RS1 with the aim of delineating a neutral pathway to INT4 that passes through the intermediate sequences identified by our path-finding algorithm (INT1, INT2, and INT3). While the first three mutations collectively lowered AIP-ligase activity by only ~four fold, the fourth mutation caused a marked reduction. The subsequent mutations generated sequences that exhibited lower levels of AIP-ligase activity, including some sequences that showed activities just above the 10% threshold. The lower activities of these intermediates may result from the adoption of an ensemble of RNA folds, most of which are inactive, as observed for “neutral” intermediates between the class III ligase and HDV ribozymes (61). PPP-ligase function emerged after 26 mutations, at which point there was also a significant rise in AIP-ligase activity. This is likely due to the creation of a stable, catalytic structure resembling CS3 that exhibits promiscuity. All intermediates that exhibit PPP-ligation on this path are part of the CS3 cluster. The appearance of PPP-ligase activity only in mutants that are close to CS3 suggests that CS3 may be far from being optimal for PPP ligation; if so, further exploration of the sequence space surrounding CS3 may reveal more active sequences. As the new CS3-like fold also catalyzed AIP-ligation, CS3 represents a distinct solution to AIP-ligation, in addition to functioning as a PPP-ligase. Our results also show that although RS1 is 28 mutations from CS3, sequences with exclusive AIP-ligase activity may be found in close proximity to PPP-ligases.

To understand how the evolution of RNA structure correlates with the evolution of catalytic function, we used computational structure prediction to follow the structural changes along the quasi-neutral mutational path from RS1 to CS3. We found that new intermediate structures were formed along the mutational path through the formation or disruption of single base pairs, one at a time. The structures could be grouped into seven distinct structural folds that include an RS1-like fold, a CS3-like fold, and five new folds connecting these two (Fig. 6*B* and *SI Appendix*, Fig. S9). While structural changes are more or less gradual with a notable absence of base-pairing between nucleotides of the constant (black) and variable (blue and green) regions in most structures (*SI Appendix*, Fig. S9 *A*–*W*), we observed a sudden and significant change in the overall fold after the 23rd mutation. The CS3-like fold of Int23, which emerged suddenly due to a single mutation to Int22, is created by extensive base-pairing between nucleotides from the constant and variable regions (*SI Appendix*, Fig. S9*X*). This can be considered a molecular version of punctuated equilibrium, often seen in experimental RNA evolution (42, 60). Taken together, our results provide experimental support for computational studies in the 1990s that emphasized the importance of multiple genotypes mapping on to a single structural phenotype in allowing neutral pathways to connect distinct structural folds (and functions) in RNA (42, 62). Although evolution operates on RNA sequences, selection pressures operate in the RNA structure space. Therefore, RS1 and CS3, two distinct RNAs, may be far in sequence space, but they need to traverse just five distinct structural folds to interconvert. Moreover, the existence of multiple structural phenotypes for a single functional phenotype (e.g., AIP ligation) underscores the importance of preserving fitness in the face of mutations, i.e., robustness. Robustness has been proposed to be critical for evolution as it allows mutational excursions on the fitness landscape and consequently, chance encounters with new functions. The mutational path between RS1 and CS3, outlined in this work supports the crucial role of robustness in enhancing the evolvability of a system (39, 40).

## Discussion

We have demonstrated that ribozymes that catalyze RNA ligation using prebiotic phosphorimidazolide substrates (AIP-ligase) may be evolved to ligase ribozymes that utilize triphosphate substrates (PPP-ligase). The ability of an AIP-ligase ribozyme to morph into a different structure with the ability to ligate both PPP- and AIP-substrates is consistent with the possibility that a primitive state of RNA-catalyzed RNA assembly using prebiotically relevant phosphorimidazolide substrates transitioned to an intermediate stage where ribozymes used triphosphate substrates for RNA assembly, thus setting the stage for the later transition to modern protein-catalyzed RNA synthesis using triphosphate substrates. The PPP-ligase sequence reported here adds to the list of ligase ribozymes that catalyze PPP-ligation. Our new PPP-ligase is distinct from most previous examples in that it catalyzes PPP-ligation on an external template, which would be critical for a ribozyme to be able to carry out general RNA assembly processes. This is in contrast to, for example, the class I ligase, where the template is supplied by a region in the ribozyme itself, and the triphosphate group is on the ribozyme 5′ terminus, which reacts with a 2′/3′-hydroxyl group of the “substrate” (19).

A remarkable feature of the evolution of the new PPP-ligase from the prior AIP-ligase is the dramatic shift in the folded structure of the ribozyme. Given that the RS1- and CS3-catalyzed ligation reactions are quite similar except for their leaving groups (two-aminoimidazole vs. pyrophosphate), one might have expected this change to require only a minor readjustment of the ribozyme structure. In reality, it appears that access to the new PPP-ligase function required a change to a radically different RNA fold. The creation of new folds to perform apparently similar functions, as opposed to minor adjustments to a binding site or active site, has been observed during the directed evolution of aptamers and ribozymes (28–33), consistent with the idea that RNA fitness landscapes are rugged such that smooth structural morphing between different functional states is not generally possible. We were surprised to find that the new PPP-ligase is promiscuous, in that it can catalyze ligation with AIP- and PPP-substrates, even though it was selected only for PPP-ligation. Why directed evolution resulted in a new structure that supports both the newly selected function, while also retaining the old function is unclear. Active site promiscuity has been observed previously in functional RNAs found in nature and evolved artificially, so one possibility is that ribozyme active sites are inherently likely to be nonspecific and, therefore, promiscuous (63–67). An alternative possibility is that the observed promiscuity might arise from the ability of the CS3 sequence to adopt two distinct catalytic folds corresponding to PPP- and AIP-ligation. However, CS3 does not fold into the parental RS1-like structure, so the dual functionality cannot result from an ability to fold into both the old and new structures. If the AIP-ligase activity is due to a distinct folded structure, that structure would have to have acquired AIP-ligase activity in the absence of selection, which seems unlikely. Furthermore, the only two ribozyme sequences known to populate two distinct catalytic structures were designed by extensive sequence engineering (43, 44), making it unlikely that CS3’s promiscuity is derived from structural plasticity. We, therefore, favor a model in which the active site of the CS3 ribozyme is itself less specific, perhaps through an ability to bind both the 2AI and the pyrophosphate leaving groups. Regardless of the underlying mechanism, the promiscuity of the PPP-ligase makes it an interesting example of a potential intermediate in the transition from nonenzymatic RNA assembly to RNA assembly with more biologically relevant substrates. Ribozyme promiscuity could have been an important driver of evolutionary innovations in an RNA World by providing opportunities for divergent functions to arise within one sequence, with subsequent evolutionary optimization following gene duplication events (38, 43, 68, 69).

We outlined a mutational path that connects the parental RS1 ligase with the evolved CS3 ligase, where each intermediate differs from its predecessor by a single mutation, and where all intermediate sequences retain at least some ligase activity. This is the third example where two distinct catalytic RNA folds are connected by a neutral path (43, 44). The existence of such a neutral path implies both the existence of a very large number of sequences that support a single function (robustness) as well as a smaller number of sequences that support both functions (promiscuity). The quasi-neutral path demonstrates the many-to-one mapping of 1) structure and function, 2) sequence and function, 3) sequence and structure, 4) function and structure, and 5) function and sequence. These redundancies in the relationship between genotype and structural and functional phenotypes in RNA make a strong case for RNA as a highly evolvable molecule.

Due to the high dimensionality of the RNA sequence space, the quasi-neutral path we identified between RS1 and CS3 is likely just one of many neutral paths that collectively constitute a neutral network bridging these two catalytic functions. In the neutral pathway outlined in this work, the sequence intermediates between the source sequence (RS1) and target sequence (CS3) show lower activities than RS1 and CS3, which would make a step-wise transition between these two functions difficult in a biological system with low mutation rates. Mutations that disrupt the overall fold or the catalytic apparatus of RS1 are likely to explain the reduced activities of these intermediate sequences. It is possible that neutral networks with more active intermediate states may be revealed through additional high-throughput sequencing and computational approaches (37, 70).

One puzzling feature of our selection experiment is that the new CS3 ligase differs by many mutations (28 out of 40 mutagenized sites) from the parental sequence. Sequences with 28 or more mutations constituted only 4 × 10^−11^ of the initial doped library, corresponding to roughly 4 × 10^4^ sequences. Even if we assume that all 28 mutations were not essential, so that the new ribozyme could have emerged from the pool of sequences with, for example, 25 or more mutations, the available pool would still correspond to less than 2 × 10^7^ sequences, whereas most novel ribozymes have emerged in previous selection experiments with a frequency of less than 10^−10^ random sequences. A possible explanation for this apparent discrepancy is that the new ribozyme first emerged from a less active sequence with fewer mutations that was present in the original doped library. Additional mutations, conferring enhanced activity, could have arisen during the amplification stages of the first few rounds of selection. However, these hypothesized ancestral sequences must be extremely rare such that we did not detect them by high-throughput sequencing. Nevertheless, this large jump from the parent sequence is not unprecedented. In prior studies, new aptamer sequences, isolated from in vitro evolution, were found at mutational distances that were farther from the parent sequence than was expected from the level of mutagenesis and the composition of the starting library (30, 31). In a landmark study, Curtus and Bartel found that >80% of all ribozymes selected from a library, generated by mutagenizing an existing ribozyme at 11% per position, were ≥12 mutations from it, when >95% of all sequences in this library were expected to be within 12 mutations. >8% of these new ribozymes were >20 mutations from the parent, underscoring the need for RNA sequences to escape existing folds to acquire new functions (33).

Ribozyme-catalyzed RNA assembly using triphosphate building blocks would have been evolutionarily advantageous even in the early phases of the RNA World due to the greater stability of PPP-substrates to hydrolysis compared to AIP-substrates. As RNA assembly reactions with PPP-substrates yield pyrophosphate as a side-product, a self-sustaining cycle may have arisen in which phosphorimidazolide substrates were converted to triphosphate substrates by the attack of pyrophosphate. Such a cycle may have facilitated the transition from prebiotic phosphorimidazolides to more biotic triphosphates as preferred substrates for enzyme-catalyzed RNA assembly. Since biological RNA assembly occurs via the polymerization of triphosphorylated monomers, and not via oligomer ligation, we speculate that PPP-ligase ribozymes may have evolved to utilize NTPs—a feat that has already been accomplished in the laboratory through directed evolution (23–26). Ribozyme-catalyzed RNA synthesis from NTPs may have, therefore, set the stage for protein polymerases to co-opt NTPs as substrates for RNA assembly. Alternatively, the first protein enzymes to catalyze RNA assembly could have been ligases that used triphosphorylated oligomers as substrates. Ligases are expected to be simpler catalysts than polymerases as they do not need to bind monomeric substrates. Instead, oligonucleotide substrates can use base-pairing to bind to RNA templates. Furthermore, ligase enzymes do not need to be as active as polymerases since fewer phosphodiester bond-forming steps are required to assemble an RNA of any given length. Consequently, ligases are expected to be more abundant in both RNA and protein sequence space and are likely to have played an important role in the early stages of modern biology (71). Indeed, protein enzymes that catalyze RNA ligation using triphosphorylated RNA oligonucleotides have been identified using laboratory evolution (72).

Regardless of the exact biochemical events that led to the emergence of modern biology, the results outlined here help us build a more complete model to connect the past and present of RNA assembly. According to this model, ribonucleotides activated with reactive groups like two-aminoimidazole generated a collection of short oligomers via polymerization. The same chemistry enabled these oligomers to get copied via the polymerization of AIP-mononucleotides and ligation of AIP-oligonucleotides. These RNA assembly reactions were then vastly improved with the emergence of ribozyme ligases and polymerases that used AIP-substrates. AIP-ligases and polymerases that acquired the ability to use more stable PPP-substrates were selected. Later, when protein polymerases evolved, possibly via intermediate ribonucleoproteins (RNPzymes), they would have been able to use PPP-substrates in the form of NTPs.

## Materials and Methods

The sequences of DNA and RNA oligonucleotides used in this study are listed in *SI Appendix*, Table S6. RNA pools and individual ligase ribozymes were prepared by in vitro transcription of PCR-generated dsDNA templates. RNA substrates were either purchased from Chemgenes or IDT. Phosphorimidazolide RNA substrates were prepared using standard activation procedures and purified using reverse-phase HPLC (9). Details about materials used in this study and experimental procedures followed are provided in *SI Appendix*.

## Supplementary Material

Appendix 01 (PDF)

## Data Availability

All study data are included in the article and/or *SI Appendix*. Codes used in this study can be found at the lab GitHub site: https://github.com/szostaklab/proj_ppp_ribozyme_evolution/ (73).
