# Supplementary material for: Evolution of the substrate specificity of an RNA ligase ribozyme from phosphorimidazole to triphosphate activation
Source: Proc Natl Acad Sci U S A. 2024 Sep 13;121(38):e2407325121. doi: 10.1073/pnas.2407325121 (PMC11420214; doi:10.1073/pnas.2407325121)
Supplement: Supplementary file 1 — Appendix 01 (PDF) [file pnas.2407325121.sapp.pdf]

PNAS

Supporting Information for

Evolution of the substrate specificity of an RNA ligase ribozyme from phosphorimidazole to triphosphate activation

Saurja DasGupta[a,b,c,1,2,3], Zoe Weiss[a,b,d,4], Collin Nisler[e,f], and Jack W. Szostak[a,b,c,3,5,6]

[a]Department of Molecular Biology, Center for Computational and Integrative Biology, Massachusetts General Hospital, Boston, MA 02114.
[b]HHMI, Massachusetts General Hospital, Boston, MA 02114.
[c]Department of Genetics, Harvard Medical School, Boston, MA 02115.
[d]Department of Molecular and Cellular Biology, Harvard University, Cambridge, MA 02138.
[e]HHMI, The University of Chicago, Chicago, IL 60637.
[f]Department of Chemistry, The University of Chicago, Chicago, IL 60637.

[1]Present address: Department of Chemistry and Biochemistry, University of Notre Dame, Notre Dame, IN 46556.
[2]Present address: Department of Biological Sciences, University of Notre Dame, Notre Dame, IN 46556.
[3]To whom correspondence may be addressed. Email: sdasgupta@nd.edu or jwszostak@uchicago.edu.
[4]Present address: Harvard/MIT MD-PhD Program, Harvard Medical School, Boston, MA 02115.
[5]Present address: HHMI, The University of Chicago, Chicago, IL 60637.
[6]Present address: Department of Chemistry The University of Chicago, Chicago, IL 60637.

CONTENTS

**1.**	**Materials and Methods**	**S3-S8**
1.1	Materials	S3
1.2	RNA preparation and substrate activation	S3
1.3	*In vitro* evolution of triphosphate ligase ribozymes	S3-S4
1.4	Ligation assays	S4
1.5	Library preparation for high-throughput sequencing	S5
1.6	Bioinformatic analysis of high-throughput sequencing data	S5
1.7	Determination of regiospecificity of the bond between CS3 and PPP-Lig	S5
1.8	Secondary structure determination by SHAPE	S5-S6
1.9	Small-angle X-ray scattering analysis	S6
1.10	Molecular Modeling and Simulation	S6-S7
1.11	Identification of the quasi-neutral pathway between RS1 and CS3	S7-S8
**2.**	**Supplementary Tables 1-6**	**S9-S18**
**3.**	**Supplementary Figures 1-9**	**S19-S30**
**4.**	**References**	**S31**

1. MATERIALS AND METHODS

1.1 Materials

All chemical reagents were purchased from Sigma, unless otherwise specified. The hydrochloride salt of 1-ethyl-3-(3-dimethylaminopropyl) carbodiimide was purchased from Alfa Aesar. The hydrochloride salt of 2-aminoimidazole was purchased from Combi-Blocks, Inc. Enzymes were purchased from New England Biolabs unless mentioned. SYBR Gold Nucleic Acid Gel Stain was purchased from ThermoFisher Scientific. 100% ethanol was purchased from Decon Laboratories, Inc. QIAquick PCR purification kits were purchased from Qiagen. The Sequagel-UreaGel concentrate and diluent system for denaturing polyacrylamide gels was purchased from National Diagnostics. Oligonucleotides used in this work are listed in Supplementary Table S6 and were purchased from Integrated DNA Technologies (IDT) except for PPP-Lig and PPP-LigB, which were purchased from Chemgenes.

1.2 RNA preparation and substrate activation

Ribozyme constructs were prepared by *in vitro* transcription of PCR-generated dsDNA templates containing 2′-*O*-methyl modifications at the last two nucleotides of the template strand to reduce transcriptional heterogeneity at the 3′ end of the RNA.(1) Each 1 ml transcription reaction contained 40 mM Tris-HCl (pH 8), 2 mM spermidine, 10 mM NaCl, 25 mM $MgCl_2$, 10 mM dithiothreitol (DTT), 30 U/mL RNase inhibitor murine, 2.5 U/mL thermostable inorganic pyrophosphatase (TIPPase), 4 mM of each NTP, 30 pmol/mL DNA template, and 1U/µL T7 RNA Polymerase and were incubated at 37°C for 3 h. DNA template was DNase I-digested (5U/mL at 37° C for 30 min) and the RNA was extracted with phenol-chloroform-isoamyl alcohol (Invitrogen), ethanol precipitated, and purified by 10% denaturing PAGE. Ligation templates, FAM-labeled primers, and ssDNA were purchased from Integrated DNA Technologies.

CS3 variants with modified 5′ chemistries (5′P and 5′OH) were generated by the splinted ligation of three RNA oligonucleotide pieces (Table S6). The first piece contained either a 5′PPP, 5′P, or a 5′OH modification. The second and third pieces were 5′-monophosphorylated to enable ligation by T4 RNA ligase 2. 1.2 nmol of each piece were incubated with 0.8 nmol each of the two DNA splints at 90 °C for 3 min followed by a 10 min incubation at 30 °C. This was followed by the addition of 1U/ µL RNA ligase 2 and 1X T4 RNA ligase buffer. The 20 µL reaction was incubated at 30 °C for 2 h, the RNA was cleaned up using Zymo Oligo Clean and Concentrator kit according to vendor protocol, and purified by 10% denaturing PAGE.

The 5′-monophosphorylated oligonucleotide corresponding to the substrate sequence (Table S6) was activated by as previously described.(2) Briefly the 5′-monophosphorylated oligonucleotide was incubated with 0.2 M 1-ethyl-3-(3 dimethylaminopropyl) carbodiimide (HCl salt) and 0.6 M 2-aminoimidazole (HCl salt, pH adjusted to 6) in aqueous solution for 2 h at room temperature, followed by four or five washes with 200 µL water per wash in Amicon Ultra spin columns (3kDa cutoff).This was followed by reverse phase analytical HPLC purification using a gradient of 98% to 75% 20 mM TEAB (triethylamine bicarbonate, pH 8) versus acetonitrile over 40 min.

1.3 *In vitro* evolution of triphosphate ligase ribozymes

The selection library was derived from the most active AIP-ligase (called RS1) identified from an earlier selection.(2) The 40 nt region in RS1 that constituted the library region in that selection was subjected to mutagenesis, where each nucleotide was doped at 21%. At each position the probability of retaining the parental nucleotide is 79%, and the other three nucleotides occurred with a 7% probability, each. The 21%-doped ssDNA library was purchased from IDT. 1.2 nmol ssDNA was converted to dsDNA by reverse transcription. The ssDNA was annealed with 1.2 nmol PCR_Rvs_primer (Table S6) and incubated with 2 mM dNTP mix, 2 mM DTT, 1X Protoscript buffer, and 5U/ µL ProtoScript II reverse transcriptase at 42 °C for 4 h. The reaction products were

purified using QIAquick PCR purification kit. The RNA library used for the first round of selection was obtained by transcribing the dsDNA as described above.

The selection strategy for isolating ribozymes capable of 5′-triphosphate RNA ligation involved partitioning active from inactive sequences by streptavidin bead capture. The partially randomized RNA library (1 µM) was incubated at room temperature with an RNA template (1.1 µM), 100 mM Tris-HCl, pH 8, 250 mM NaCl, and substrate, PPP-LigB (1.2 µM). The amount of RNA library used in each round, the incubation times, and the Mg^{2+} concentrations for each round are listed in Table S1. Reactions were quenched by adding 200 mM EDTA, 200 µM Quench_Oligo1 (a deoxyoligonucleotide complementary to the substrate) and 20 µM Quench_Oligo2 (a deoxyoligonucleotide complementary to the 3′ end of the ribozyme-primer construct) (Table S6). The reaction mixture was heated to 95 °C for 2 min, cooled to RT, then incubated with MyOne Streptavidin C1 Dynabeads (1 mg/100 pmol RNA). Before use, Dynabeads were prepared by washing twice with buffer 1 (1 M NaCl, 1 mM EDTA, 10 mM Tris pH 7.6, 0.2% Tween-20), once with buffer 3 (25 mM NaOH, 50 mM NaCl, 1 mM EDTA), then twice more with buffer 1. 1 mL buffer was used for washing 1 mg beads. Beads were blocked to reduce non-specific RNA retention by tumbling in 1 mL buffer 1 containing 10 µg/mL tRNAs for 1 h. Following blocking, beads were washed twice with buffer 1, and the quenched reaction mixture was added to the beads in the presence of buffer 1. To immobilize biotinylated RNAs, the solution was tumbled for 30 min. The beads were washed with buffer 1 twice, then tumbled with buffer 2 (8 M urea, 1 M NaCl, 1 mM EDTA, 10 mM Tris pH 7.6, 0.1% Tween-20) for 20 min, twice, then tumbled with buffer 3 for 5 min, twice. After three washes with buffer A and removal of liquids, 100 µL of the elution mix (95% formamide, 10 mM EDTA) was added to the beads and the beads were incubated at 65 °C for 6 min to elute the immobilized RNA from the beads. The eluted RNA was ethanol precipitated by adding 200 µL water, 50 µL sodium acetate (pH 5.2), 2 µL glycogen (20 mg/ml; RNA grade - ThermoFisher Scientific) and 900 µL 100% cold ethanol and storing at -80 °C for 30 min. The pellet was washed with 70% ethanol and air dried. All washes were with 1 mL buffer and a Dynal magnetic tube rack was used for magnetic separation.

The pellet was resuspended in water and reverse transcribed with ProtoScript II reverse transcriptase and reverse transcription primer, RT_primer (Table S6) at 42 °C for 3 h, as described before. The enzyme was heat inactivated at 80 °C for 5 min and the product was purified with QIAquick PCR purification kit. The purified product was directly used as input for a PCR with primers, PCR_Fwd_primer and PCR_Rvs_primer (Table S6). The PCR cycle was: 1) 95 °C for 2 min; 2) 10 cycles of 94 °C for 30 s, 54 °C for 1 min., and 72 °C for 1 min.; 3) 72 °C for 10 min. The PCR product was purified using QIAquick PCR purification kit and 100 ng of the PCR product was further amplified in a second PCR as above for 8 cycles. These steps were taken to minimize amplification due to the presence of RT_primer, which may complete with PCR_Rvs_primer during PCR. The dsDNA from this PCR was transcribed to generate the input RNA library for the next round and was also subjected to the three sequencing PCRs to generate the corresponding sequencing library for high-throughput sequencing (see ‘Library preparation for high-throughput sequencing’).

1.4 Ligation assays

Ligation reactions contained 1 µM ribozyme, 1.2 µM RNA template, and 2 µM RNA substrate in 100 mM Tris-HCl pH 8.0, 300 mM NaCl, and either 10 mM $MgCl_2$ for AIP-Lig or 100 mM $MgCl_2$ for PPP-Lig/PPP-LigB/P-LigB/P-Lig/OH-Lig. Aliquots were quenched with 5 volumes of quench buffer (8 M urea, 100 mM Tris-Cl, 100 mM boric acid, 100 mM EDTA) and analyzed on a 10% denaturing PAGE. Gels were stained using SYBR Gold and imaged on an Amersham Typhoon RGB instrument (GE Healthcare), Scans were analyzed in ImageQuant IQTL 8.1. Kinetic data were nonlinearly fitted to the modified first order rate equation, $y = A(1 - e^{-kx})$, where A represents the fraction of active complex, k is the first order rate constant, x is time, and y is the fraction of ligated product in GraphPad Prism 9.

1.5 Library preparation for high-throughput sequencing

RNA pools were sequenced after every round of selection. To prepare the selected pools for sequencing, sequencing adaptors were introduced via three rounds of PCR with different primer sets (Table S6), using the dsDNAs from each pool that were used to generate the RNA pool for the next round. In the first round, 25 ng dsDNA was amplified in a 100 μL PCR containing 0.5 μM primers, SeqPCR_primer1 and SeqPCR_primer2, by Taq DNA polymerase for 4 cycles. The PCR product was purified using QIAquick PCR Purification Kit (Qiagen), and then further amplified for 7 cycles in the second round of PCR containing 40 ng dsDNA, 0.5 μM primers, SeqPCR_primer3 and SeqPCR_primer4. The purified product of the second PCR was amplified in a 50 μL reaction containing 1 unit of Q5 Hot Start High-Fidelity DNA Polymerase, 100 ng of ds DNA, 1X Q5 buffer, 0.2 μM each of SR Primer for illumina and Index Primer for illumina (New England Biolabs), and 80 μM dNTPs. This third round PCR mixture was incubated at 98 °C for 30 s, followed by 6 cycles of 98 °C for 10 s, 62 °C for 15 s, and 72 °C for 20 s, and finally, 72 °C for 2 min. The PCR product was purified using QIAquick PCR Purification Kit and then on a 1.4% agarose gel. Purified DNAs were extracted from agarose gels by Freeze N' Squeeze gel extraction spin columns (Bio-Rad). The DNA was purified from the residual dye and concentrated using an Oligo Clean & Concentrator Kit (Zymo Research) and sequenced in an illumina MiSeq instrument.

1.6 Bioinformatic analysis of high-throughput sequencing data

Sequencing reads from each round were pre-processed using Python scripts as follows. Sequences were first filtered for the presence of the two predefined 8 nt sequences that flank the variable region, and only sequences that passed this filter were subject to quality control. Sequences that satisfied the quality score threshold of 20 for each nucleotide position ($Q \geq 20$) with less than one percent error were trimmed to the 40 nt variable region.(3) Trimmed sequences were dereplicated using a custom script and clustered using the Clustal Omega algorithm.(4, 5)

The relative abundances of CS1-CS5, the peak sequences of the five most abundant clusters, were plotted across rounds to generate Fig. 2C. To generate Fig. 5A, we counted the number of reads for sequences at different mutational distances from the parent AIP-ligase, RS1, and represented their relative abundances for rounds 1-6 in a heat map. Only sequences with ≥100 reads are shown. Similarly, Fig. 5B was generated by plotting the fractional abundances of sequences (with ≥100 reads) in the CS3 cluster across rounds 1-6. To generate Fig. S6A, we counted the frequency with which each of the four nucleotides occur at a given position in the CS3 peak sequence and plotted the probability fraction against nucleotide number. This reveals nucleotide conservation within the CS3 cluster. Fig. S6B was generated using the WebLogo online server (https://weblogo.berkeley.edu/logo.cgi).

1.7 Determination of regiospecificity of the bond between CS3 and PPP-Lig

~4 pmol of the purified ligated product obtained from an overnight ligation reaction between CS3 and PPP-Lig was digested with 20 U of RNase R (Lucigen) in a 10 μL reaction in the presence of 1X RNase R buffer at 50 ºC for 1 h. The reaction was quenched by adding 0.3 μL of 0.5 M EDTA and the enzyme was deactivated by heating at 95 ºC for 3 min. 10 μL of quench buffer (8 M urea, 100 mM Tris-Cl, 100 mM boric acid, 100 mM EDTA) was added to the heat-inactivated reaction, and the reaction was analyzed by 10% denaturing PAGE.

1.8 Secondary structure determination by SHAPE

SHAPE probing of CS3 was performed using a protocol reported in Walton *et al.*, 2020.(9) The sequence used for SHAPE probing (CS3_SHAPE) consisting of 5′ and 3′ SHAPE cassettes is included in Table S6. Briefly, 100 pmol CS3_SHAPE was folded in 100 mM Tris-HCl, pH 8, 250 mM NaCl, 10 mM Mg^{2+} and divided into 'modification' and 'control' tubes. SHAPE reagent, 1M7 (in DMSO) was added to a final concentration of ~40 mM in the 'modification' tube and only DMSO was added to the 'control' tube. Both modified and unmodified RNAs were reverse transcribed

using 40 pmol of a 5′ FAM-labeled primer (SHAPE_RT_primer) and Superscript III reverse transcriptase (Invitrogen) (Table S6). Reverse transcription of 30 pmol unmodified RNAs in the presence of each of the four ddNTPs and 25 pmol SHAPE_RT_primer was used to generate sequencing lanes. Reactions and quenched and analyzed by 10% denaturing PAGE. Normalized SHAPE reactivity was calculated by first excluding the most reactive nucleotide position and then dividing reactivities at each position by the average of the 10% most reactive positions. Normalized SHAPE reactivities were used to constrain secondary structure prediction in the RNAstructure program.(6)

1.9 Small-angle X-ray scattering analysis

SAXS data were collected at the SIBYLS beamline at the Advanced Light Source (Berkeley, CA) as previously described.(7-9) For CS3, two concentrations were analyzed: 0.5 mg ml^{-1} and 1 mg ml^{-1}. For RS1, three concentrations were analyzed: 0.5, 1, and 2 mg ml^{-1}. Data were collected in 10 mM $MgCl_2$, 200 mM NaCl, and 200 mM Tris pH 7.5 at 20˚C. The X-ray wavelength was set to 1.127 Å with a sample-to-detector distance of 2,100 mm, which gives scattering vectors (q) ranging from 0.01 Å-1 to 0.4 $Å^{-1}$. The SAXS flow cell was coupled to an Agilent 1260 Infinity HPLC system using a Shodex 802.5 SEC column equilibrated with the running buffer as indicated above with a flow rate of 0.65 ml min^{-1}. For SAXS measurements, 2 second X-ray exposures were collected during a 25-minute elution. Buffer subtraction was performed with buffer matched controls. For CS3, the 0.5 mg ml^{-1} sample provided the best Guinier fit and was used for subsequent analysis. For RS1, the 1 mg ml^{-1} sample provided the best Guinier fit and was used for subsequent analysis.

The radius of gyration (R_g) was calculated for each buffer subtracted frame using the Guinier approximation in the program RAW.(10) Final merged SAXS profiles obtained by integration of multiple frames at the elution peak were used for further analyses. The volume of correlation (V_c) was used to estimate molecular weight,(11) and the pair distribution function [P(r)] was used to calculate maximum inter-particle distances (Table S5).(12) Molecular envelopes for each ribozyme were calculated using DENSS(13) considering q<0.2. Ten molecular surfaces were generated and averaged for each ribozyme. Scattering intensities were generated from molecular models saved during the molecular dynamics simulations (described below) and compared to experimental intensities using FOXS.(14) The model with the lowest chi-square value for RS1 and CS3 as predicted by FOXS was fit in the averaged molecular envelope using ChimeraX.(15) For RS1, the best predicted fit to the experimental data included a single model, while for CS3 the best predicted fit to the experimental data required a two-state model.

1.10 Molecular Modeling and Simulation

Rosetta's FARFAR2(16) method was used to generate initial molecular models for RS1 and CS3. The top 10 scoring structures were generated using secondary structure constraints derived from SHAPE and with high resolution optimization after fragment assembly using the -minimize_rna flag. For RS1, an initial set of 10 models was generated without the linker or primer region present, and the second model from this set was chosen for simulation due to the more open placement of the 5′ overhang relative to the stem-loop region (segment 1). A second set of 10 models was generated without the 5′ overhang but with the linker and primer region present. The structure of the primer, linker, and four bases upstream of the linker region were saved from the highest scoring model of this set of predictions (segment 2). Segment 2 was aligned to segment 1 based on the backbone atoms of the overlapping four 5′ bases of segment 2, which correspond to the final 3′ bases of segment 1. The coordinates of the aligned segment 2 were saved without the overlapping four 5′ bases and were joined with the coordinates of segment 1, resulting in the complete structure of RS1. For CS3, a first set of 10 models were generated that consisted of the tail and two stem-loop regions without the linker and primer, and from these model 4 was selected (segment 1). A second set of 10 models was generated that contained only the second stem-loop

region, the linker, and the primer, and from these the first model was selected (segment 2). Segment 2 was aligned to segment 1 by overlapping the final 4 5′ bases of segment 1 with the first four 3′ bases of segment 2 via their backbone atoms. The coordinates of the aligned segment 2 were saved, and the coordinates of segment 1 without the second stem-loop region were saved. These two were joined to form the complete structure of CS3.

The starting structures of RS1 and CS3 used for all-atom molecular dynamics simulations were generated as described above. The VMD psfgen, solvate, and autoionize plugins were used to build both systems.(17) TIP3P water boxes were made large enough to prevent interaction with periodic images, and 50 mM NaCl was added to both systems. Both the 5' and 3' ends of RS1 and CS3 were capped with an -OH group. Simulations were run using NAMD 2.14(18) with the CHARMM36 force field and the TIP3P explicit model of water.(19) A cutoff of 12 Å was used for van der Waals interactions, and long-range electrostatic forces were computed using the Particle Mesh Ewald method with a grid point density of >1 $Å^{-3}$. The SHAKE algorithm was used with a timestep of 2 fs. The NpT ensemble was used at 1 atm with a hybrid Nose-Hoover Langevin piston method. The temperature was set to 300 K. Both systems were minimized for 5,000 steps, followed by 100,000 steps of equilibration in which the backbone atoms were constrained. Finally, constraints were lifted, and the system was equilibrated for 50 ns. For both RS1 and CS3, the NAMD collective variables module was used to apply weak restraints on the interactions of terminal G-C and A-U stem base pairs. Root mean square deviation (RMSD) was calculated by aligning the non-hydrogen backbone atoms to the initial frame of each simulation after constrained equilibration.

To create models for the RS1 and CS3 structures including the template and substrate in a post-reaction state (where ribozyme, primer, and substrate are continuous sequences), FARFAR2 was used to generate the structure of the template/substrate/primer duplex, and the best scoring model was selected. For both RS1 and CS3, the four bases at the 3' end (AAGG) of this duplex were aligned to the AAGG of the initial ribozyme structure used for the 50 ns simulations described above. The template/substrate/primer duplex structure was saved without the 5' AAGG, and the RS1 and CS3 ribozyme structures (without the unpaired 5' end) were combined with the template/substrate duplex to generate the final complete model of the unbound state (Fig. S8). Both structures were solvated, ionized, and simulated for 200 ns using identical parameters and protocols as described above.

To create a model for the substrate bound state of CS3, FARFAR2 was used to generate a structure with the SHAPE-derived secondary structure constraints as shown in Fig. 3A. The best scoring result was selected, and this structure was aligned to the final frame of the 200 ns simulation of the CS3 unbound state simulation based on residues C57 and G58. The structure of the first stem of the 200 ns simulation was saved (up to C54) and combined with the structure of the second stem interacting with the substrate/template duplex generated from FARFAR2 to generate the final complete model of the CS3 bound state (Fig. S8). This structure was solvated, ionized, and simulated for 100 ns using identical parameters and protocols as described above.

1.11 Identification of the quasi-neutral pathway between RS1 and CS3

We employed Dijkstra's algorithm, a common algorithm for finding the shortest paths using an adjacency matrix, to identify a path between RS1 and CS3. We used an adjacency matrix of RNA sequences obtained from all rounds of selection. The adjacency matrix was constructed by finding the Hamming distance between each pair of sequences and connecting the sequences with a distance equal to one mutation. We then ran the shortest path finding algorithm on this adjacency matrix (single mutation matrix) and looked for the path to the most similar sequence. The algorithm used for the shortest path finding was based on the breadth-first search algorithm, which is known to be efficient and accurate for finding the shortest paths in graphs.

We implemented the algorithm in Python. Our path represents the graph using a dictionary, where the keys are the nodes, and the values are the lists of adjacent nodes. The function inputs the graph, start node, and goal node and returns the shortest path between them. The function first initializes an empty list, which will track nodes that have been visited. It then uses a while loop which continues until there are no nodes left in the queue. It checks the adjacency list of nodes in the graph dictionary and iterates over each adjacent node. If the adjacent node is the goal node, the function returns the new path. Otherwise, the function moves on to the next adjacent node. If the goal node is never found, the function returns a 0, indicating that no path was found. The code begins by looping over all sequences 1 mutation from CS3 and searching for a path from RS1 to each sequence. If no complete paths are found, it starts again by looping over all sequences 2 mutations from CS3. Again, if no paths are found, it tries sequences 3 mutations from CS3 and so on until a complete path is found. Once a path is found, the code calculates the similarity between the mutant and the original CS3 using a sequence matching function. Finally, the code prints the path from RS1 to the mutant and the similarity score between the mutant and the original CS3.

We first chose the path which ended with the sequence most similar to CS3 (INT3 in Table S3). INT3 is only 2 mutations from RS1. We then repeated the same algorithm in reverse, with CS3 as the start sequence and INT3 as the goal sequence. As expected, a compete path was not generated but the sequence closest to INT3 was revealed as INT4 which is 24 mutations from INT3 and 3 mutations from CS3. This provided us with the nearest complete path from both directions and allowed us to experimentally fill in the gaps. We designed sequence variants to complete the single-step mutational path and by experimental trial and error filled in the gaps with mutant sequences that exhibited ligase activity above our activity threshold (rate acceleration of >10% of the target sequence, CS3, i.e., 10-fold over background for AIP-ligation and 100-fold over background for PPP-ligation).

2. SUPPLEMENTARY TABLES

Table S1. Reaction parameters for each round of selection and sequencing outputs. Selection was started with 3 nmol RNA to sample a large number of sequences, which was decreased to 50 pmol in the final round (R6). Reaction stringency was increased by reducing reaction times and Mg^{2+} concentrations. This resulted in sequence enrichment across rounds with a marked decrease in sequence diversity in round 4 (from ~66% unique sequences to ~7% unique sequences).

	R1	R2	R3	R4	R5	R6
**Scale (pmol)**	3000	1000	300	100	50	50
**Reaction time (min)**	180	180	60	30	30	5
**[Mg^{2+}] (mM)**	100	50	50	20	20	5
**No. of high-quality sequences**	1793335	1824368	1238531	1959238	1553726	1515844
**% Unique sequences**	97.6	94.3	66.1	7.2	7.2	6.3

Table S2. CS3 cluster sequences with >100 reads in round 6. Sequences in the CS3 cluster, sorted in decreasing order of their abundance. The top 3 sequences cover ~80% of the cluster population. Nucleotide positions that differ from the peak sequence CS3 are indicated in boldface. For population dynamics across rounds, see Figure 5B.

No.	Library Sequence (40 nt, 5′ → 3′)<br>1 5 10 15 20 25 30 35 40	Fractional abundance
1.	ACGGGTGGGTAATCTAGTGTCCGCGGAATAGAACGAAACA	0.540
2.	ACG**A**GTGGGTAATCTAGTGTCCGCGGAATAGAACGAAACA	0.200
3.	ACGGGTGGGTAATCTAGTGTC**T**GCGGAATAGAACGAAACA	0.038
4.	ACGGGTGGGTAATCT**G**GTGTCCGCGGAATAGAACGAAACA	0.025
5.	ACGGGTGGGTAA**C**CTAGTGTCCGCGGAATAGAACGAAACA	0.024
6.	ACGGGTG**T**GTAATCTAG**C**GTCCGCGGAATAGAACGAAACA	0.022
7.	ACGGGTG**AA**TAATCTAG**C**GTCCGCGGAATAGAACGAAACA	0.019
8.	ACGGGTGGGTAATCTAGTGTCCGCGGAATAGAACGA**T**ACA	0.013
9.	ACGGGTG**A**GTAATCTAG**C**GTCCGCGGAATAGAACGAAACA	0.011
10.	ACGGGTG**AA**TAATCTAGTGTCCGCGGAATAGAACGAAACA	0.009
11.	ACG**A**GTGGGTAATCTAGTGTC**T**GCGGAATAGAACGAAACA	0.008
12.	ACGGGTG**AA**TAATCTA**A**TGTCCGCGGAATAGAACGAAACA	0.006
13.	ACG**A**GTGGGTAATCT**G**GTGTCCGCGGAATAGAACGAAACA	0.006
14.	ACGGGTGGG**C**AATCTAGTGTCCGCGGAATAGAACGAAACA	0.005
15.	ACG**A**GTGGGTAATCTAGTGTCCGCGGAATAGAACGA**T**ACA	0.005
16.	ACGGGTGGGTAATCTAG**C**GTCCGCGGAATAGAACGAAACA	0.004
17.	ACGGGTGGGTAATCTAGTGTCCGCGG**G**ATAGAACGAAACA	0.004
18.	ACGGGTG**A**GTAATCTAGTGTCCGCGGAATAGAACGAAACA	0.004
19.	ACGGGTG**AA**TAATCTAGTGTC**T**GCGGAATAGAACGAAACA	0.002
20.	ACGGGTGGGTAA**C**CTAGTGTC**T**GCGGAATAGAACGAAACA	0.002
21.	ACG**A**GTGGG**C**AATCTAGTGTCCGCGGAATAGAACGAAACA	0.002
22.	ACG**A**GTGGGTAATCTAGTGTCCGCGG**G**ATAGAACGAAACA	0.002
23.	ACGGGTGGGTAATCTAGTGTCCGCGGAA**G**AGAACGAAACA	0.002
24.	ACGGGTGGGTAATCTAGT**T**TCCGCGGAATAGAACGAAACA	0.002
25.	ACGGGTGGGTAATCTAGTGTCCGCGGA**G**TAGAACGAAACA	0.001
26.	ACG**A**GTGGGTAA**C**CTAGTGTCCGCGGAATAGAACGAAACA	0.001
27.	ACGGG**C**GGGTAATCTAGTGTCCGCGGAATAGAACGAAACA	0.001
28.	ACGGGTGGGTAATCTAGTGTCCGCGGAATAGAACGAAAC**G**	0.001
29.	ACGGGTGGGTAATCTAGTGTCCGCGGAATAGAACGAAAC**C**	0.001
30.	ACGGGTGGGTAATCTAGTGTCCGCGGAATAGAACGA**G**ACA	0.001
31.	ACGGGTGGGTAATCTAGTGTCCGCGGAATAGAACGAA**G**CA	0.001
32.	ACGGGTG**T**GTAATCTAGTGTCCGCGGAATAGAACGAAACA	0.001
33.	ACGGGTGGGTAATCTAGTGTCCGCGGAATAGA**G**CGAAACA	0.001
34.	ACGGGTGGGTAATCTAGTG**C**CCGCGGAATAGAACGAAACA	0.001

No.	Library Sequence (40 nt, 5′ → 3′)<br>1 5 10 15 20 25 30 35 40	Fractional abundance
36.	ACG**T**GTGGGTAATCTAGTGTCCGCGGAATAGAACGAAACA	0.001
37.	ACGGGTGGGTAATCTAGTGTCCGCGGAATAG**G**ACGAAACA	0.001
38.	ACGGGTGGGTAATCTAGTGTC**T**GCGGAATAGAACGA**T**ACA	0.001
39.	ACGGGTGG**T**TAATCTAGTGTCCGCGGAATAGAACGAAACA	0.001
40.	ACG**A**GTGGGTAATCTAG**C**GTCCGCGGAATAGAACGAAACA	0.001
41.	ACGGGTGGGTAATC**C**AGTGTCCGCGGAATAGAACGAAACA	0.001
42.	ACGGGTGGGTA**G**TCTAGTGTCCGCGGAATAGAACGAAACA	0.001
43.	A**A**GGGTGGGTAATCTAGTGTCCGCGGAATAGAACGAAACA	0.001
44.	**G**CGGGTGGGTAATCTAGTGTCCGCGGAATAGAACGAAACA	0.001
45.	ACGGGTGGGTAATCTAGTGTCCGCG**T**AATAGAACGAAACA	0.001
46.	ACGGGTGGGTAATCTAGTGTCCGCGGAATAGAACG**G**AACA	0.001
47.	ACGGGTGGGTAATCTA**A**TGTCCGCGGAATAGAACGAAACA	0.001
48.	ACGGGTGGGTAATCTAGTGTCCG**A**GGAATAGAACGAAACA	0.001
49.	ACGGGTGGGTAATCTAGTGTCCGCGGAATAGAACG**C**AACA	0.001
50.	ACGGGTGGGTAATCTAGTGTC**A**GCGGAATAGAACGAAACA	0.001
51.	ACGGGTGGGTAATCTAGTGTCCGCGGAAT**G**GAACGAAACA	0.001
52.	ACGGGTGGGTAATCT**G**GTGTCCGCGGA**G**TAGAACGAAACA	0.001
53.	ACGGGTGGGTAATCTAGTGTC**G**GCGGAATAGAACGAAACA	0.001
54.	ACGGGTGGGTAATCTAGTGT**T**CGCGGAATAGAACGAAACA	0.001
55.	ACGGGTGGGTAATCT**G**GTGTCCGCGGAATAGAACGA**T**ACA	0.001
56.	ACGGGTGGGTAATCTAGTGTC**T**GCGG**G**ATAGAACGAAACA	0.001
57.	ACGGGTGGGTAA**C**CT**G**GTGTCCGCGGAATAGAACGAAACA	0.001
58.	ACGGGTG**AA**TAATCTAGTGT**T**CGCGGAATAGAACGAAACA	0.001
59.	ACG**A**GTGGGTAATCTAGT**T**TCCGCGGAATAGAACGAAACA	0.001
60.	ACGGGTGGGTAATCTAGTGTCCGCGGAATAGAACGAAAC**T**	0.001
61.	ACG**A**GTGGGTAATCTAGTGTCCGCGGAA**G**AGAACGAAACA	0.001
62.	ACGGGT**A**GGTAATCTAGTGTCCGCGGAATAGAACGAAACA	0.001
63.	ACGGGTGGGTAATCTAGTGTCC**T**CGGAATAGAACGAAACA	0.001
64.	ACGGGTGGGTAA**C**CTAGTGTCCGCGGAATAGAACGA**T**ACA	0.001
65.	ACGGGTG**AA**TAATCTAGTG**C**CCGCGGAATAGAACGAAACA	0.001
66.	ACGGGTGGGTAATCTAGTGTCCGCGGAATAGAAC**T**AAACA	0.001
67.	ACGGGTGGGTAATCTAGTGTCC**A**CGGAATAGAACGAAACA	0.001
68.	ACGGGTGGGTAATCTAGTGTCCGCGG**T**ATAGAACGAAACA	0.001

Table S3. Intermediate sequences between RS1 and CS3, identified by mining high-throughput sequencing data from rounds 1-6. INT4 (indicated by an asterisk) resembles CS3 (3 mutations from it) and is 25 mutations from RS1. The mutational space between INT3 and INT4 was filled experimentally to generate Figure 6 and Table S4.

**Name**	**Library Sequence (40 nt, 5′ → 3′)**
	**1 5 10 15 20 25 30 35 40**
RS1	GAAUGCUGCCAACCGUGCGGGCUAAUUGGCAGACUGAGCU
INT1	GAAUGCUGCCAACCGUGCGGGCUAAUU**C**GCAGACUGAGCU
INT2	GAAUGCUGCCAACC**U**UGCGGGCUAAUU**C**GCAGACUGAGCU
INT3	GAAUGCUGCCAACC**U**UGCGGGCUAAUU**A**GCAGACUGAGCU
INT4*	**ACGA**G**UG**GC**U**AA**U**C**UA**G**U**G**UC**C**GCGGAAUAG**GAC**GA**A**A**C**A**
INT5	**ACGA**G**UG**G**GU**AA**U**C**UA**G**U**G**UC**C**GCGGAAUAG**GAC**GA**A**A**C**A**
INT6	**ACGG**G**UG**G**GU**AA**U**C**UA**G**U**G**UC**C**GCGGAAUAG**GAC**GA**A**A**C**A**
CS3	**ACGG**G**UG**G**GU**AA**U**C**UA**G**U**G**UC**C**GCGGAAUAGA**AC**GA**A**A**C**A**

Table S4. Mutational pathway connecting RS1 to CS3. Point mutations to RS1 are highlighted in bold. PPP-Ligase activity emerges in Sequence 26, which is accompanied by a marked increase in AIP-ligation. The two-dimensional fitness landscape for ligase function containing these sequences is shown in Figure 6.

Name	Library Sequence (40 nt, 5′ → 3′)	k_{obs} (h^{-1}) AIP	k_{obs} (h^{-1}) PPP
	1 5 10 15 20 25 30 35 40		
RS1	GAAUGCUGCCAACCGUGCGGGCUAAUUGGCAGACUGAGCU	6.38 ± 0.60	-
Int1	GAAUGCUGCCAACC**U**UGCGGGCUAAUUGGCAGACUGAGCU	1.78 ± 0.24	-
Int2	GAAUGCUGCCAACC**U**UGCGGGCUAAUU**A**GCAGACUGAGCU	2.78 ± 0.36	-
Int3	GAA**A**GCUGCCAACC**U**UGCGGGCUAAUU**A**GCAGACUGAGCU	1.59 ± 0.23	-
Int4	GAA**A**GCUGC**U**AACC**U**UGCGGGCUAAUU**A**GCAGACUGAGCU	0.30 ± 0.08	-
Int5	GAA**A**GCUGC**U**AACC**UA**GCGGGCUAAUU**A**GCAGACUGAGCU	0.19 ± 0.04	-
Int6	GAA**A**GCUGC**U**AACC**UA**GCGG**C**CUAAUU**A**GCAGACUGAGCU	0.20 ± 0.05	-
Int7	GAA**A**GCUGC**U**AACC**UA**GCGG**C**CUAAUU**A**GCAGACU**A**AGCU	0.103 ± 0.01	-
Int8	GAA**A**GCUGC**U**AACC**UA**GCGG**C**CUAAUU**A**G**A**AGACU**A**AGCU	0.134 ± 0.12	-
Int9	GAA**A**GCUGC**U**AA**U**C**UA**GCGG**C**CUAAUU**A**G**A**AGACU**A**AGCU	0.20 ± 0.03	-
Int10	GAA**A**GCUGC**U**AA**U**C**UA**GCGG**C**C**G**AAUU**A**G**A**AGACU**A**AGCU	0.30 ± 0.06	-
Int11	GAA**A**GCUGC**U**AA**U**C**UA**GCGG**C**C**G**AAUU**A**G**A**AGACU**A**AGC**A**	0.12 ± 0.04	-
Int12	GAA**A**GCUGC**U**AA**U**C**UA**GCGG**C**C**G**AAUU**A**G**A**AGACU**A**A**A**C**A**	0.38 ± 0.05	-
Int13	GAA**A**GCUGC**U**AA**U**C**UA**GCGG**C**C**G**AAUU**A**G**A**AGAC**GA**A**A**C**A**	0.61 ± 0.08	-
Int14	GAA**A**GCUGC**U**AA**U**C**UA**GCGG**C**C**G**AAUU**A**G**AG**GAC**GA**A**A**C**A**	0.66 ± 0.19	-
Int15	GAA**A**GCUGC**U**AA**U**C**UA**GCGG**C**C**G**AAUU**AUAG**GAC**GA**A**A**C**A**	0.26 ± 0.04	-
Int16	GAA**A**GCUGC**U**AA**U**C**UA**GCGG**C**C**G**AAU**AAUAG**GAC**GA**A**A**C**A**	0.11 ± 0.05	-
Int17	GAA**A**GC**G**GC**U**AA**U**C**UA**GCGG**C**C**G**AAU**AAUAG**GAC**GA**A**A**C**A**	0.09 ± 0.05	-
Int18	GAA**A**GC**G**GC**U**AA**U**C**UA**GCGG**C**C**G**AA**GAAUAG**GAC**GA**A**A**C**A**	0.12 ± 0.04	-
Int19	GAA**A**GC**G**GC**U**AA**U**C**UA**GCGG**C**C**G**A**GGAAUAG**GAC**GA**A**A**C**A**	0.09 ± 0.05	-
Int20	GAA**A**GC**G**GC**U**AA**U**C**UA**GCGG**C**C**GCGGAAUAG**GAC**GA**A**A**C**A**	1.00 ± 0.05	-
Int21	GAA**A**GC**G**GC**U**AA**U**C**UA**GCG**UC**C**GCGGAAUAG**GAC**GA**A**A**C**A**	0.25 ± 0.04	-
Int22	GAA**A**GC**G**GC**U**AA**U**C**UA**G**U**G**UC**C**GCGGAAUAG**GAC**GA**A**A**C**A**	0.46 ± 0.01	-
Int23	GAA**A**G**UG**GC**U**AA**U**C**UA**G**U**G**UC**C**GCGGAAUAG**GAC**GA**A**A**C**A**	0.21 ± 0.02	-
Int24	GA**GA**G**UG**GC**U**AA**U**C**UA**G**U**G**UC**C**GCGGAAUAG**GAC**GA**A**A**C**A**	0.14 ± 0.05	-
Int25	G**CGA**G**UG**GC**U**AA**U**C**UA**G**U**G**UC**C**GCGGAAUAG**GAC**GA**A**A**C**A**	0.19 ± 0.05	-
Int26	**ACGA**G**UG**GC**U**AA**U**C**UA**G**U**G**UC**C**GCGGAAUAG**GAC**GA**A**A**C**A**	1.03 ± 0.15	0.009 ± 0.002
Int27	**ACGA**G**UG**G**GU**AA**U**C**UA**G**U**G**UC**C**GCGGAAUAG**GAC**GA**A**A**C**A**	0.86 ± 0.13	0.004 ± 0.001
Int28	**ACGG**G**UG**G**GU**AA**U**C**UA**G**U**G**UC**C**GCGGAAUAG**GAC**GA**A**A**C**A**	0.45 ± 0.01	0.005 ± 0.001
CS3	**ACGG**G**UG**G**GU**AA**U**C**UA**G**U**G**UC**C**GCGGAAUAGA**AC**GA**A**A**C**A**	1.06 ± 0.01	0.012 ± 0.003
	1 5 10 15 20 25 30 35 40		

Table S5. SAXS Parameters for RS1 and CS3.

SAXS parameters	RS1	CS3
R_g (Å) [from P(r)]	36.07 ± 1.81	37.93 ± 0.94
R_g (Å) [from Guinier]	30.33 ± 1.34	31.38 ± 3
D_{max} (Å)	149.0	123.0
Molecular Weight (kDa)	32.8	36.4

Table S6. Oligonucleotides used in this work. In the ribozyme sequences, the variable region are highlighted in blue, the T7 promoter sequence is shown in purple, the U_6 linker is shown in italics, the primer sequence is underlined, and the 3′ terminal nucleotide possessing the nucleophile for the ligation reaction is shown in boldface. 5′ and 3′ SHAPE cassettes are highlighted in green. Oligonucleotides were either purchased from Integrated DNA Technologies (IDT) or Chemgenes or generated enzymatically by *in vitro* transcription (IVT) of dsDNA templates. AIP-Lig and MeIP-Lig were generated by incubating P-Lig with EDC and 2-aminoimidazole (2AI) and 2-methylamidazole (2MeI), respectively (See 'RNA preparation and substrate activation' in Materials and Methods).

No.	Oligo Name	Sequence (5′→3′)	Type	Source
1.1	RS1	GACUCACUGACACAGAUCCACUCACGGACAGCGGAAUGCUGCCAACCGUGCGGGCUAAUUGGCAGACUGAGCUCGCUGUCC*UUUUUU*GGCUAAG**G**	RNA	IVT
1.2	RS1_doped21_DNA (Mutagenesis at 21% at each nucleotide position: 79% WT nucleotide and 7% of the other three) Note: The sequence is written according to IDT specifications	TAATACGACTCACTATAGACTCACTGACACAGATCCACTCACGGACAGCG(N1:07077907)(N2:79070707)(N2)(N3:07070779)(N1)(N4:07790707)(N3)(N1)(N4)(N4)(N2)(N2)(N4)(N4)(N1)(N3)(N1)(N4)(N1)(N1)(N1)(N4)(N3)(N2)(N2)(N3)(N3)(N1)(N1)(N4)(N2)(N1)(N2)(N4)(N3)(N1)(N2)(N1)(N4)(N3)CGCTGTCC*TTTTTT*GGCTAAG**G**	DNA	IDT
2.1	Template	GCGGUGGUCCUUAGCC	RNA	IDT
2.2	Template+1	UGCGGUGGUCCUUAGCC	RNA	IDT
2.3	Template+2	AUGCGGUGGUCCUUAGCC	RNA	IDT
2.4	Template+3	AAUGCGGUGGUCCUUAGCC	RNA	IDT
2.5	Template+4	GAAUGCGGUGGUCCUUAGCC	RNA	IDT
2.6	Template+5	GGAAUGCGGUGGUCCUUAGCC	RNA	IDT
2.7	Template+6	CGGAAUGCGGUGGUCCUUAGCC	RNA	IDT
2.8	Template+7	GCGGAAUGCGGUGGUCCUUAGCC	RNA	IDT
2.9	Template+8	UGCGGAAUGCGGUGGUCCUUAGCC	RNA	IDT
2.10	Template_U8A	GCGGUGGACCUUAGCC	RNA	IDT
2.11	Template_C9G	GCGGUGGUGCUUAGCC	RNA	IDT
3.1	PPP-LigB	(5′-triphosphate) - ACCACCGCAUUCCGCA - (3BioTEG)	RNA	Chemgenes
3.2	PPP-Lig	(5′-triphosphate) - ACCACCGCAUUCCGCA	RNA	Chemgenes
3.3	AIP-Lig	(5′-phosphoro-2-aminoimidazole) - ACCACCGCAUUCCGCA	RNA	Activation of P-Lig
3.4	P-LigB	(5′-monophosphate) - ACCACCGCAUUCCGCA - (3BioTEG)	RNA	IDT
3.5	P-Lig	(5′-monophosphate) - ACCACCGCAUUCCGCA	RNA	IDT
3.6	HO-Lig	(5′-hydroxyl) - ACCACCGCAUUCCGCA	RNA	IDT
3.7	MeIP-Lig	(5′-phosphoro-2-methylimidazole) - ACCACCGCAUUCCGCA	RNA	Activation of P-Lig
4	RT primer	GTGCGGAATGCGGTGGTCCTT	DNA	IDT
5.1	Quench oligo 1	GTGCGGAATGCGGTGGT	DNA	IDT
5.2	Quench oligo 2	GCCTTAGCCAAAAAAGGACAGCG	DNA	IDT
6.1	PCR_Forward_primer	TAATACGACTCACTATAGACTCACTGACAC	DNA	IDT

6.2	PCR_Reverse_primer	mCmCTTAGCCAAAAAAGGACAGCG	DNA	IDT
6.3	SeqPCR_primer1	GTTCAGAGTTCTACAGTCCGACGATCCGGTAGG<br>TCCCTTAGCCAAAAAAGGACAGCG	DNA	IDT
6.4	SeqPCR_primer2	AGACGTGTGCTCTTCCGATCTGACTCACTGACA<br>CAGATCCACTCAC	DNA	IDT
6.5	SeqPCR_primer3	GTTCAGAGTTCTACAGTCCGACGATC	DNA	IDT
6.6	SeqPCR_primer4	AGACGTGTGCTCTTCCGATCT	DNA	IDT
7.1	CS1	GACUCACUGACACAGAUCCACUCACGGACAGCG<br>GACAGCCGAGAAAUGAGUGGCCUAAAUGGGAGA<br>AUGAGCUCGCUGUCC*UUUUUU*GGCUAAG**G**	RNA	IVT
7.2	CS2	GACUCACUGACACAGAUCCACUCACGGACAGCG<br>GACUGCGCGUAUGAGUGGCGGCUAAAGAGGAGA<br>AUGAGCGCGCUGUCC*UUUUUU*GGCUAAG**G**	RNA	IVT
7.3	CS3	GACUCACUGACACAGAUCCACUCACGGACAGCG<br>ACGGGUGGGUAAUCUAGUGUCCGCGGAAUAGAA<br>CGAAACACGCUGUCC*UUUUUU*GGCUAAG**G**	RNA	IVT
7.4	CS3_5't	GGACAGCGACGGGUGGGUAAUCUAGUGUCCGCG<br>GAAUAGAACGAAACACGCUGUCC*UUUUUU*GGCU<br>AAG**G**	RNA	IVT
7.5	CS3_3't	GACUCACUGACACAGAUCCACUCACGGACAGCG<br>ACGGGUGGGUAAUCUAGUGUCCGCGGAAUAGAA<br>CGAAACACGCUGUCC	RNA	IVT
7.6	CS4	GACUCACUGACACAGAUCCACUCACGGACAGCG<br>GGAUGGUGCGAACUGAGUGGGCUAAUUAGGAGA<br>AUGAGCGCGCUGUCC*UUUUUU*GGCUAAG**G**	RNA	IVT
7.7	CS5	GACUCACUGACACAGAUCCACUCACGGACAGCG<br>GGAGGGUGACAUCGUUGAGAGAGAAUGGGGAUA<br>UUGAACUCGCUGUCC*UUUUUU*GGCUAAG**G**	RNA	IVT
8.1	PPP-CS3_pc1	(5′-triphosphate) –<br>GACUCACUGACACAGAUCCACUCAC	RNA	IVT
8.2	P-CS3_pc1	(5′-monophosphate) –<br>GACUCACUGACACAGAUCCACUCAC	RNA	IDT
8.3	HO-CS3_pc1	(5′-hydroxyl) –<br>GACUCACUGACACAGAUCCACUCAC	RNA	IDT
8.4	pCS3_pc2	(5′-monophosphate) –<br>GGACAGCGACGGGUGGGUAAUCUAGUGUCCGCG<br>GAAUAGA	RNA	IDT
8.5	pCS3_pc3	(5′-monophosphate) –<br>ACGAAACACGCUGUCCUUUUUUGGCUAAGG	RNA	IDT
8.6	CS3_splint1	ACCCACCCGTCGCTGTCCGTGAGTGGATCTGTG<br>TCAG	DNA	IDT
8.7	CS3_splint2	CCAAAAAAGGACAGCGTGTTTCGTTCTATTCCG<br>CGGACAC	DNA	IDT
9.1	CS3_SHAPE	GGCCUUCGGGCCAAGACUCACUGACACAGAUCC<br>ACUCACGGACAGCGACGGGUGGGUAAUCUAGUG<br>UCCGCGGAAUAGAACGAAACACGCUGUCCUUUU<br>UUGGCUAAG**G**UCGAUCCGGTTCGCCGGAUCCAA<br>AUCGGGCUUCGGUCCGGUUC	RNA	IVT
9.2	SHAPE_RT_primer	(5′-FAM)-GAACCGGACCGAAGCCCG		
10.1	Int1	GACUCACUGACACAGAUCCACUCACGGACAGCG<br>GAAUGCUGCCAACCUUGCGGGCUAAUUGGCAGA<br>CUGAGCUCGCUGUCC*UUUUUU*GGCUAAG**G**	RNA	IVT

10.2	Int2	GACUCACUGACACAGAUCCACUCACGGACAGCG<br>GAAUGCUGCCAACCUUGCGGGCUAAUUAGCAGA<br>CUGAGCUCGCUGUCC*UUUUUU*GGCUAAG**G**	RNA	IVT
10.3	Int3	GACUCACUGACACAGAUCCACUCACGGACAGCG<br>GAAAGCUGCCAACCUUGCGGGCUAAUUAGCAGA<br>CUGAGCUCGCUGUCC*UUUUUU*GGCUAAG**G**	RNA	IVT
10.4	Int4	GACUCACUGACACAGAUCCACUCACGGACAGCG<br>GAAAGCUGCUAACCUUGCGGGCUAAUUAGCAGA<br>CUGAGCUCGCUGUCC*UUUUUU*GGCUAAG**G**	RNA	IVT
10.5	Int5	GACUCACUGACACAGAUCCACUCACGGACAGCG<br>GAAAGCUGCUAACCUAGCGGGCUAAUUAGCAGA<br>CUGAGCUCGCUGUCC*UUUUUU*GGCUAAG**G**	RNA	IVT
10.6	Int6	GACUCACUGACACAGAUCCACUCACGGACAGCG<br>GAAAGCUGCUAACCUAGCGGCCUAAUUAGCAGA<br>CUGAGCUCGCUGUCC*UUUUUU*GGCUAAG**G**	RNA	IVT
10.7	Int7	GACUCACUGACACAGAUCCACUCACGGACAGCG<br>GAAAGCUGCUAACCUAGCGGCCUAAUUAGCAGA<br>CUAAGCUCGCUGUCC*UUUUUU*GGCUAAG**G**	RNA	IVT
10.8	Int8	GACUCACUGACACAGAUCCACUCACGGACAGCG<br>GAAAGCUGCUAACCUAGCGGCCUAAUUAGAAGA<br>CUAAGCUCGCUGUCC*UUUUUU*GGCUAAG**G**	RNA	IVT
10.9	Int9	GACUCACUGACACAGAUCCACUCACGGACAGCG<br>GAAAGCUGCUAAUCUAGCGGCCUAAUUAGAAGA<br>CUAAGCUCGCUGUCC*UUUUUU*GGCUAAG**G**	RNA	IVT
10.10	Int10	GACUCACUGACACAGAUCCACUCACGGACAGCG<br>GAAAGCUGCUAAUCUAGCGGCCGAAUUAGAAGA<br>CUAAGCUCGCUGUCC*UUUUUU*GGCUAAG**G**	RNA	IVT
10.11	Int11	GACUCACUGACACAGAUCCACUCACGGACAGCG<br>GAAAGCUGCUAAUCUAGCGGCCGAAUUAGAAGA<br>CUAAGCACGCUGUCC*UUUUUU*GGCUAAG**G**	RNA	IVT
10.12	Int12	GACUCACUGACACAGAUCCACUCACGGACAGCG<br>GAAAGCUGCUAAUCUAGCGGCCGAAUUAGAAGA<br>CUAAACACGCUGUCC*UUUUUU*GGCUAAG**G**	RNA	IVT
10.13	Int13	GACUCACUGACACAGAUCCACUCACGGACAGCG<br>GAAAGCUGCUAAUCUAGCGGCCGAAUUAGAAGA<br>CGAAACACGCUGUCC*UUUUUU*GGCUAAG**G**	RNA	IVT
10.14	Int14	GACUCACUGACACAGAUCCACUCACGGACAGCG<br>GAAAGCUGCUAAUCUAGCGGCCGAAUUAGAGGA<br>CGAAACACGCUGUCC*UUUUUU*GGCUAAG**G**	RNA	IVT
10.15	Int15	GACUCACUGACACAGAUCCACUCACGGACAGCG<br>GAAAGCUGCUAAUCUAGCGGCCGAAUUAUAGGA<br>CGAAACACGCUGUCC*UUUUUU*GGCUAAG**G**	RNA	IVT
10.16	Int16	GACUCACUGACACAGAUCCACUCACGGACAGCG<br>GAAAGCUGCUAAUCUAGCGGCCGAAUAAUAGGA<br>CGAAACACGCUGUCC*UUUUUU*GGCUAAG**G**	RNA	IVT
10.17	Int17	GACUCACUGACACAGAUCCACUCACGGACAGCG<br>GAAAGCGGCUAAUCUAGCGGCCGAAUAAUAGGA<br>CGAAACACGCUGUCC*UUUUUU*GGCUAAG**G**	RNA	IVT
10.18	Int18	GACUCACUGACACAGAUCCACUCACGGACAGCG<br>GAAAGCGGCUAAUCUAGCGGCCGAAGAAUAGGA<br>CGAAACACGCUGUCC*UUUUUU*GGCUAAG**G**	RNA	IVT

10.19	Int19	GACUCACUGACACAGAUCCACUCACGGACAGCG GAAAGCGGCUAAUCUAGCGGCCGAGGAAUAGGA CGAAACACGCUGUCC*UUUUUU*GGCUAAG**G**	RNA	IVT
10.20	Int20	GACUCACUGACACAGAUCCACUCACGGACAGCG GAAAGCGGCUAAUCUAGCGGCCGCGGAAUAGGA CGAAACACGCUGUCC*UUUUUU*GGCUAAG**G**	RNA	IVT
10.21	Int21	GACUCACUGACACAGAUCCACUCACGGACAGCG GAAAGCGGCUAAUCUAGCGUCCGCGGAAUAGGA CGAAACACGCUGUCC*UUUUUU*GGCUAAG**G**	RNA	IVT
10.22	Int22	GACUCACUGACACAGAUCCACUCACGGACAGCG GAAAGCGGCUAAUCUAGUGUCCGCGGAAUAGGA CGAAACACGCUGUCC*UUUUUU*GGCUAAG**G**	RNA	IVT
10.23	Int23	GACUCACUGACACAGAUCCACUCACGGACAGCG GAAAGUGGCUAAUCUAGUGUCCGCGGAAUAGGA CGAAACACGCUGUCC*UUUUUU*GGCUAAG**G**	RNA	IVT
10.24	Int24	GACUCACUGACACAGAUCCACUCACGGACAGCG GAGAGUGGCUAAUCUAGUGUCCGCGGAAUAGGA CGAAACACGCUGUCC*UUUUUU*GGCUAAG**G**	RNA	IVT
10.25	Int25	GACUCACUGACACAGAUCCACUCACGGACAGCG GCGAGUGGCUAAUCUAGUGUCCGCGGAAUAGGA CGAAACACGCUGUCC*UUUUUU*GGCUAAG**G**	RNA	IVT
10.26	Int26	GACUCACUGACACAGAUCCACUCACGGACAGCG ACGAGUGGCUAAUCUAGUGUCCGCGGAAUAGGA CGAAACACGCUGUCC*UUUUUU*GGCUAAG**G**	RNA	IVT
10.27	Int27	GACUCACUGACACAGAUCCACUCACGGACAGCG ACGAGUGGCUAAUCUAGUGUCCGCGGAAUAGAA CGAAACACGCUGUCC*UUUUUU*GGCUAAG**G**	RNA	IVT
10.28	Int28	GACUCACUGACACAGAUCCACUCACGGACAGCG ACGAGUGGGUAAUCUAGUGUCCGCGGAAUAGAA CGAAACACGCUGUCC*UUUUUU*GGCUAAG**G**	RNA	IVT

3. SUPPLEMENTARY FIGURES

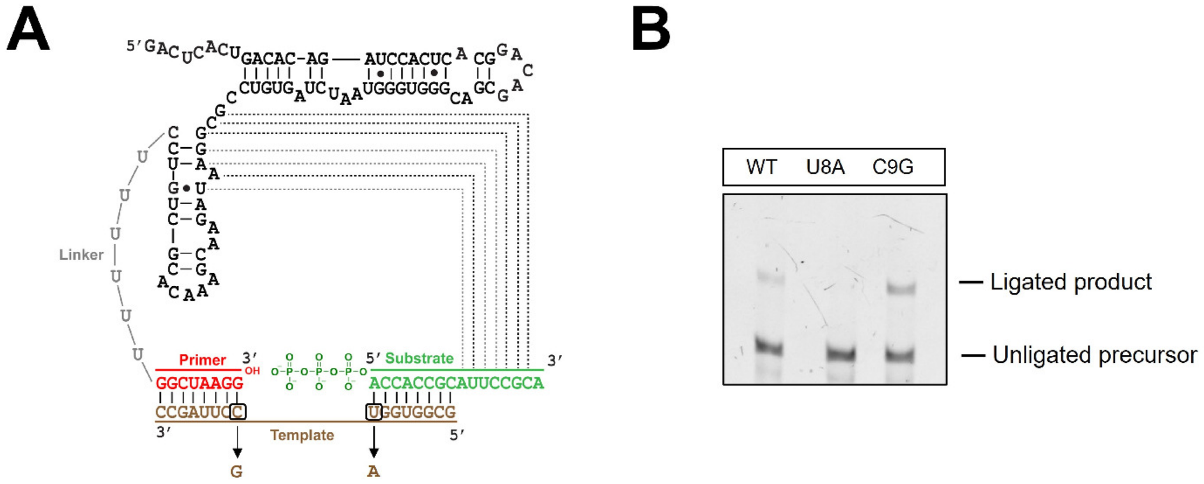

Figure S1. Effect of disrupting the ligation junction. A. CS3 in complex with the template and substrate, showing mutations in the template (U8A and C9G) that disrupt the ligation junction at the substrate and primer end, respectively. **B.** The U8A mutation, which unpairs the terminal base pair between the substrate and the template, abrogates ligation. The C9G mutation, which unpairs the terminal base pair between the primer and the template, preserves ligation. Ligation reactions contained 1 μM ribozyme, 1.2 μM RNA template, and 2 μM PPP-Lig in 100 mM Tris-HCl (pH 8.0), 300 mM NaCl, and 100 mM $MgCl_2$. Template sequences used in these experiments can be found in Table S6.

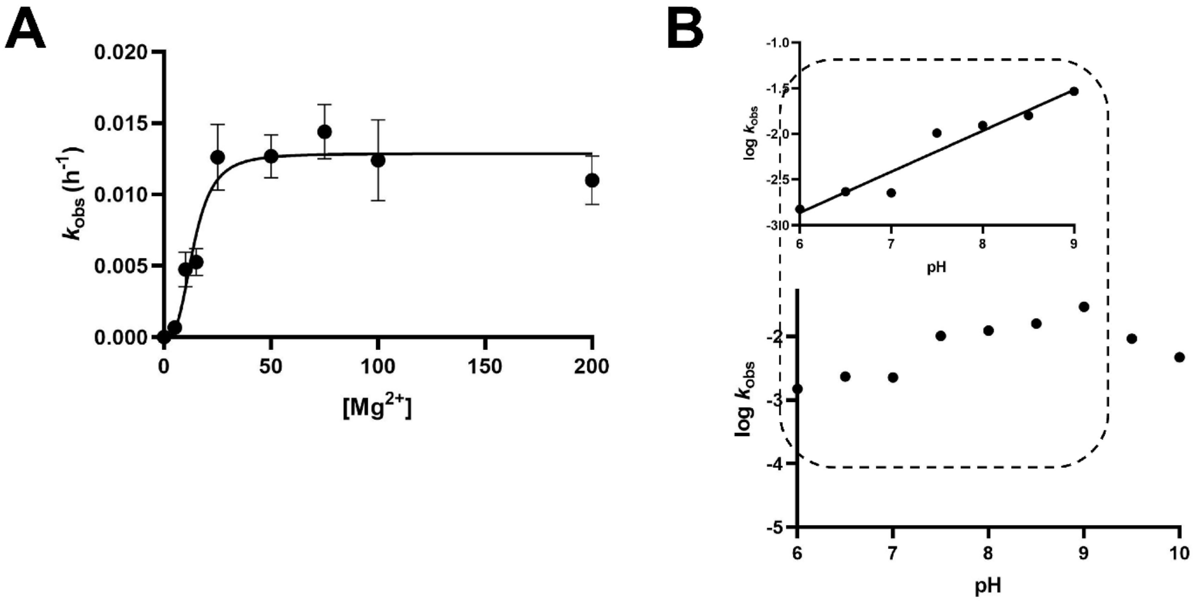

Figure S2. The effect of Mg^{2+} concentration and pH on the rates of CS3-catalyzed ligation of PPP-Lig. A. The k_{obs} value for PPP-ligation increase steeply till [Mg^{2+}] reaches 25 mM and plateaus at higher concentrations. The data is plotted to the Hill Equation and yields a $[Mg^{2+}]_{1/2}$ = 13.9 ± 1.79 mM and is consistent with the binding of ~3 Mg^{2+} ions. Ligation reactions contained 1 μM ribozyme, 1.2 μM RNA template, and 2 μM PPP-Lig in 100 mM Tris-HCl (pH 8.0), 300 mM NaCl, and the indicated amounts of $MgCl_2$. **B.** Ligation rates increase from pH 6 to pH 9 and then fall. The inset shows that the increase in ligation rate is log-linear in the range pH 6-9 with a slope of 0.45 ± 0.05. Ligation conditions were identical as in (A), except that the reactions contained 100 mM $MgCl_2$ and were performed at the indicated pH values.

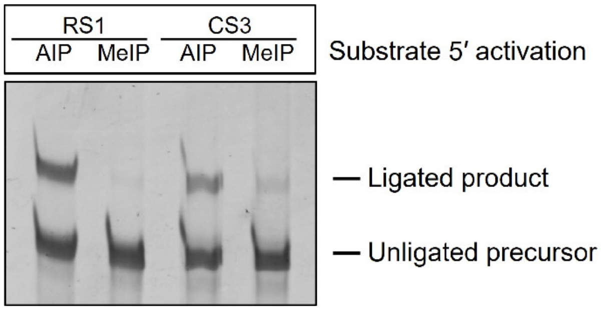

Figure S3. The effect of substrate activation on ligation by RS1 and CS3. RS1 is specific to 2-aminoimidazole-activated substrates; however, CS3 can ligate substrates activated with both 2-aminoimidazole (AIP-Lig) and 2-methylimidazole (MeIP-Lig). Ligation reactions contained 1 μM ribozyme, 1.2 μM RNA template, and 2 μM substrate in 100 mM Tris-HCl (pH 8.0), 300 mM NaCl, and 10 mM $MgCl_2$.

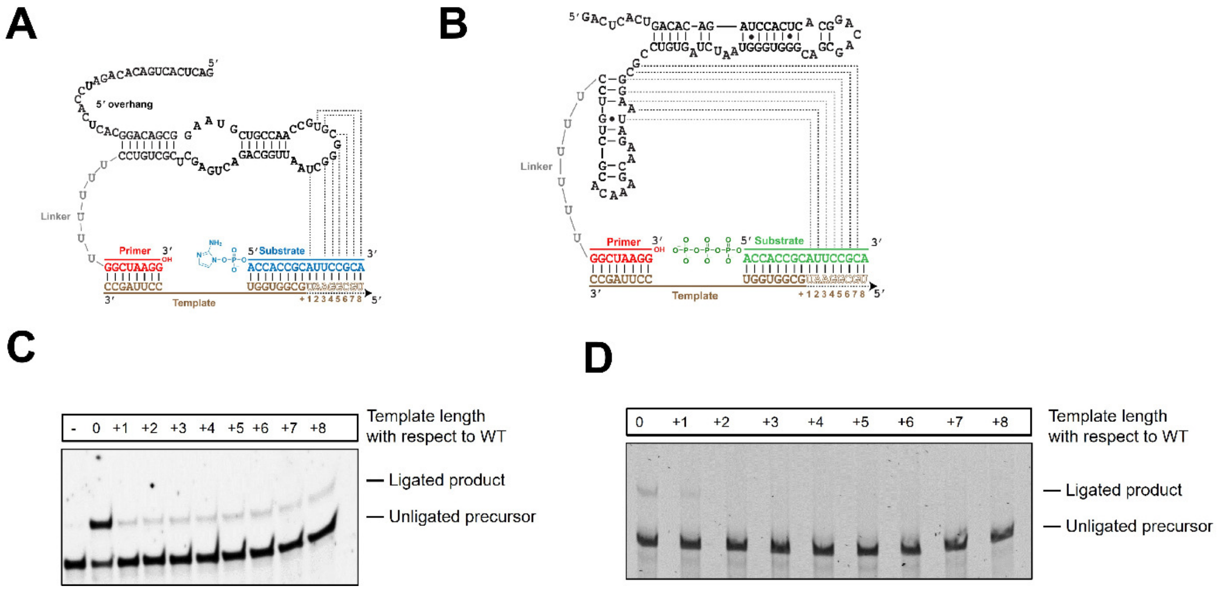

Figure S4. Progressive sequestering of the 3′ substrate overhang results in a decrease in ligation by RS1 and CS3. Extending the length of the template (brown) at its 5′ end sequesters the 3' overhang of **A.** AIP-Lig and **B.** PPP-Lig. **C.** Significant reduction in ligation is observed when the template is extended by 1 nt in the case of AIP-ligation. **D.** Extending the template by 1 nt reduces ligation for PPP-ligation, and further extension eliminates ligation. Ligation reactions contained 1 µM ribozyme, 1.2 µM RNA template, and 2 µM AI-Lig or PPP-Lig in 100 mM Tris-HCl (pH 8.0), 300 mM NaCl, and 10 mM (for AIP-ligation) or 100 mM (for PPP-ligation) $MgCl_2$. Template sequences used in these experiments can be found in Table S6.

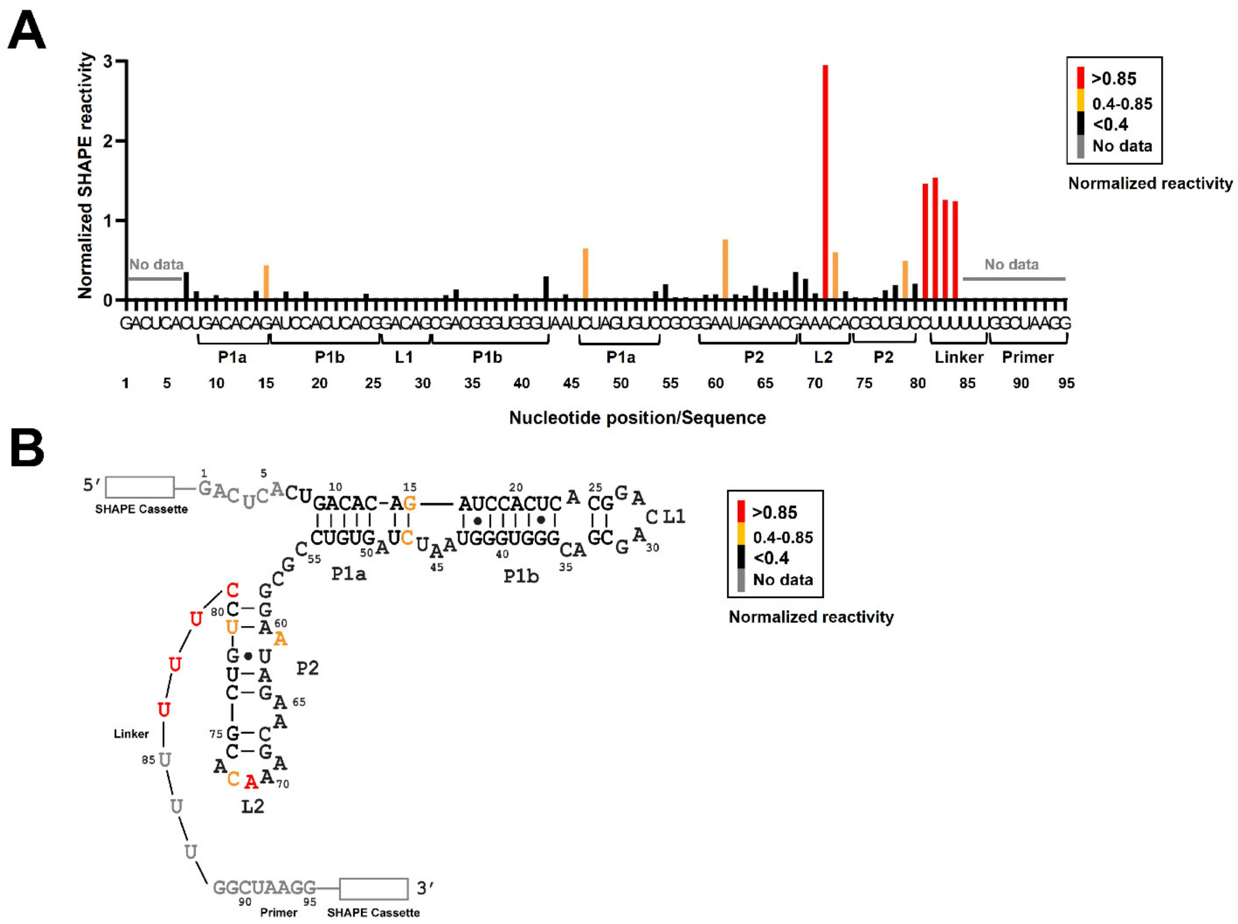

Figure S5. SHAPE analysis of CS3. Products of primer extension reactions with FAM-labeled primers after modification with 1M7 were separated on a 10% denaturing PAGE. **A.** Normalized SHAPE reactivities were plotted for each nucleotide position. High reactivity suggests flexibility within the RNA structure, while low reactivity suggests base-paired stems or interactions with distal nucleotides in its tertiary fold. **B.** Secondary structure of CS3 determined by the RNAstructure program using reactivity constraints obtained from SHAPE experiments. 5′ and 3′ SHAPE cassettes are denoted by white rectangles. Nucleotides for which no data was obtained are shown in gray. Nucleotides are colored in red, orange, and black according to their normalized SHAPE reactivities, as shown in the reactivity legend. Sequences used in SHAPE experiments can be found in Table S6.

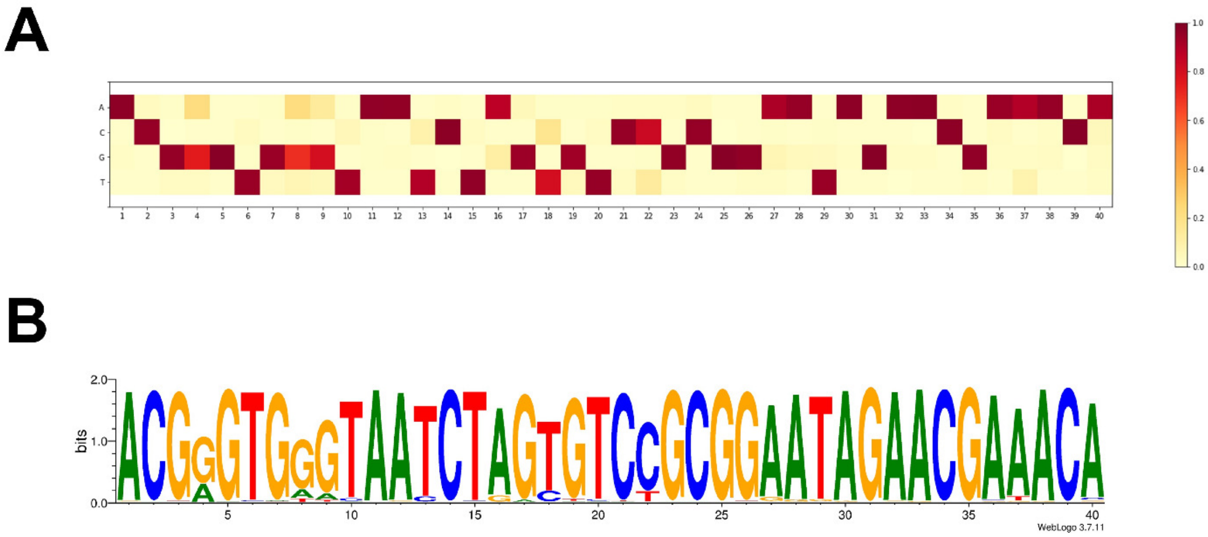

Figure S6. High degree of nucleotide conservation in the 40 nt variable region of CS3. A. Heat map showing the fractional abundances of each of the four nucleotides for each nucleotide position. **B.** A sequence logo shows the consensus sequence, depicting the relative abundances of each nucleotide for each position. The sequence logo was generated by the WebLogo online server (https://weblogo.berkeley.edu/logo.cgi).

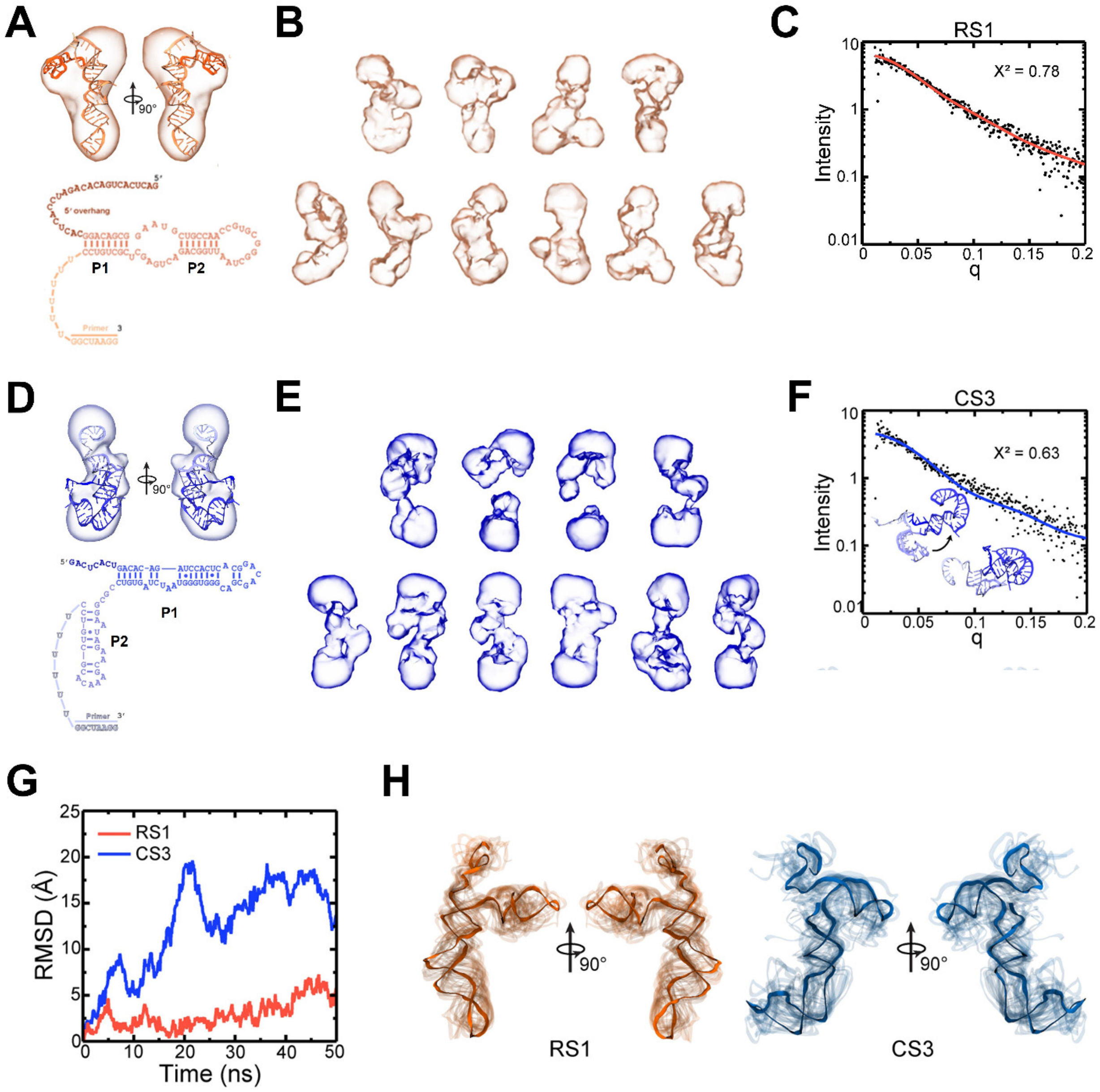

Figure S7. Structural modeling of RS1 and CS3. A. The computed molecular model of RS1 is shown fit to the calculated molecular envelope, with the color gradient of the molecular model corresponding to the secondary structure diagram. **B.** The ten surfaces shown were calculated by DENSS and averaged to give the final envelope in (A). **C.** Computed intensity profile (orange line) to the experimental SAXS profile (black dots) of the molecular model exhibiting the lowest chi-square value. **D.** The computed molecular model of CS3 is shown fit to the calculated molecular envelope, with the color gradient of the molecular model corresponding to the secondary structure diagram. **E.** The ten surfaces shown were calculated by DENNS and averaged to give the final envelope in (D). **F.** Computed intensity profile (blue line) to the experimental SAXS profile (black dots) of the molecular models exhibiting the best fit. The two models included in this calculation are shown in the inset. **G.** RMSD values shown for RS1 and

CS3 over the 50 ns equilibrations. **H.** Structures of RS1 and CS3 were aligned by their non-hydrogen backbone atoms and are shown every 2 ns in transparent backbone representation. The final frame of the simulation is shown in opaque backbone representation. The RMSD and molecular figures indicate a more dynamic structure for CS3, which agrees with the two-state fit to the SAXS data for CS3.

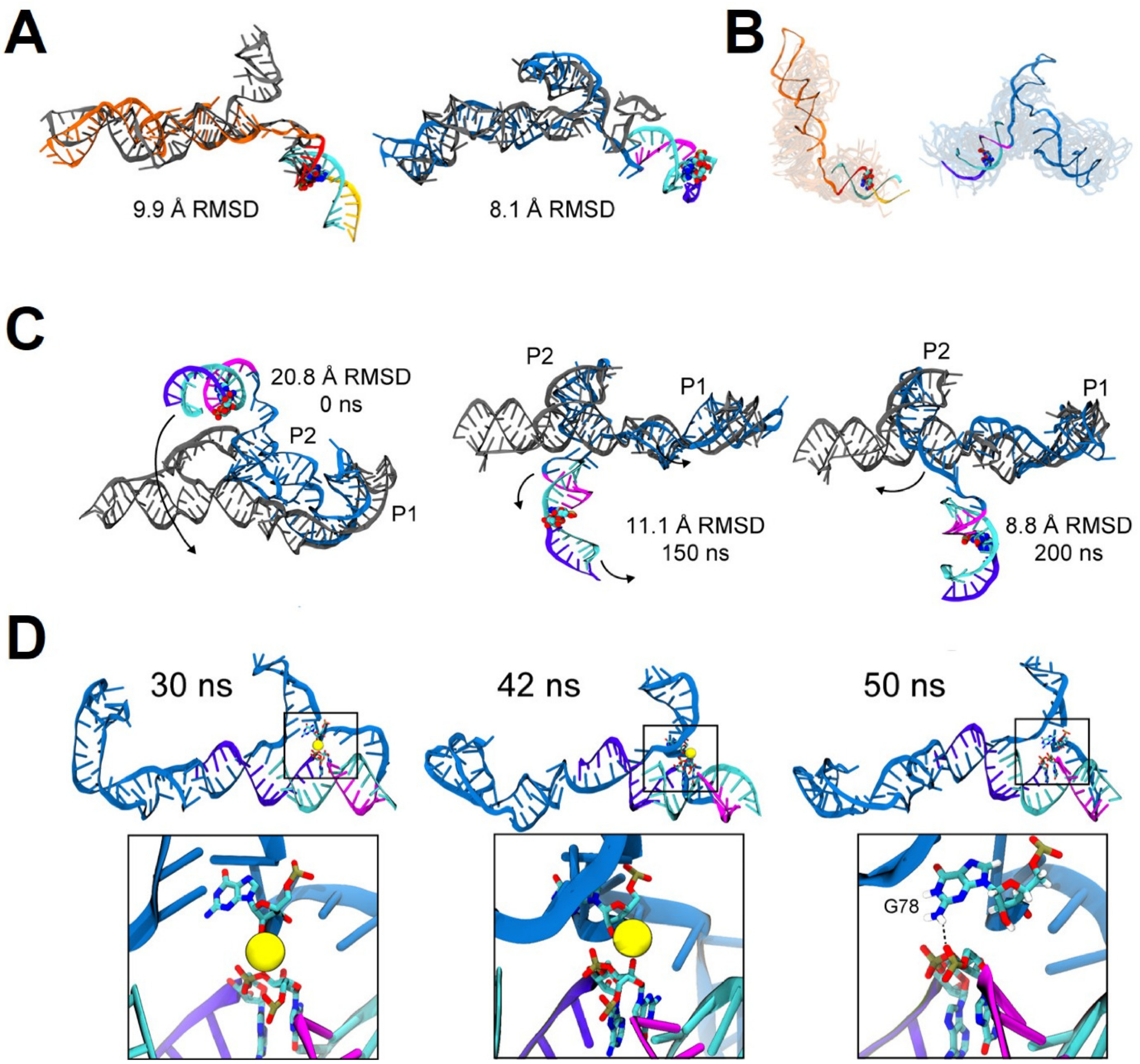

Figure S8. Structural modeling and simulation of RS1 and CS3 including the substrate and the template. A. Full-length (post-ligation) RS1 after partial equilibration (left, orange) and full-length CS3 after equilibration (right, blue) aligned to the final structure of the respective simulation from Figure S7 (grey). RMSD values are calculated based on backbone atoms of shared residues. For RS1, the ribozyme is shown in orange, the primer in red, the substrate in yellow, and the template in cyan. For CS3, the ribozyme is shown in blue, the primer in magenta, the substrate in violet, and the template in cyan. **B.** Structures of RS1 (orange) and CS3 (blue) were aligned by their non-hydrogen backbone atoms and are shown every 30 ns in transparent backbone representation. The final frame of the simulation is shown in opaque backbone representation. **C.** Final state of the CS3 structure with interaction between substrate and ribozyme (grey) aligned to the structure of the CS3 without the interaction between substrate and ribozyme at 0, 150, and 200 ns. RMSD values are calculated from backbone atoms of both stems. The colors of the unbound state structure are the same as in (A). Arrows indicate the motion of the

substrate/template duplex relative to stem P1 and P2, which results in a reduced RMSD relative to the bound state. **D.** Three snapshots showing a Na^+ (yellow sphere) coordinated with the reaction site facilitated by a residue from the base of the second stem of the ribozyme (first two frames), or a direct interaction with the reaction site by G78 (final frame). The ribozyme is shown as in (A).

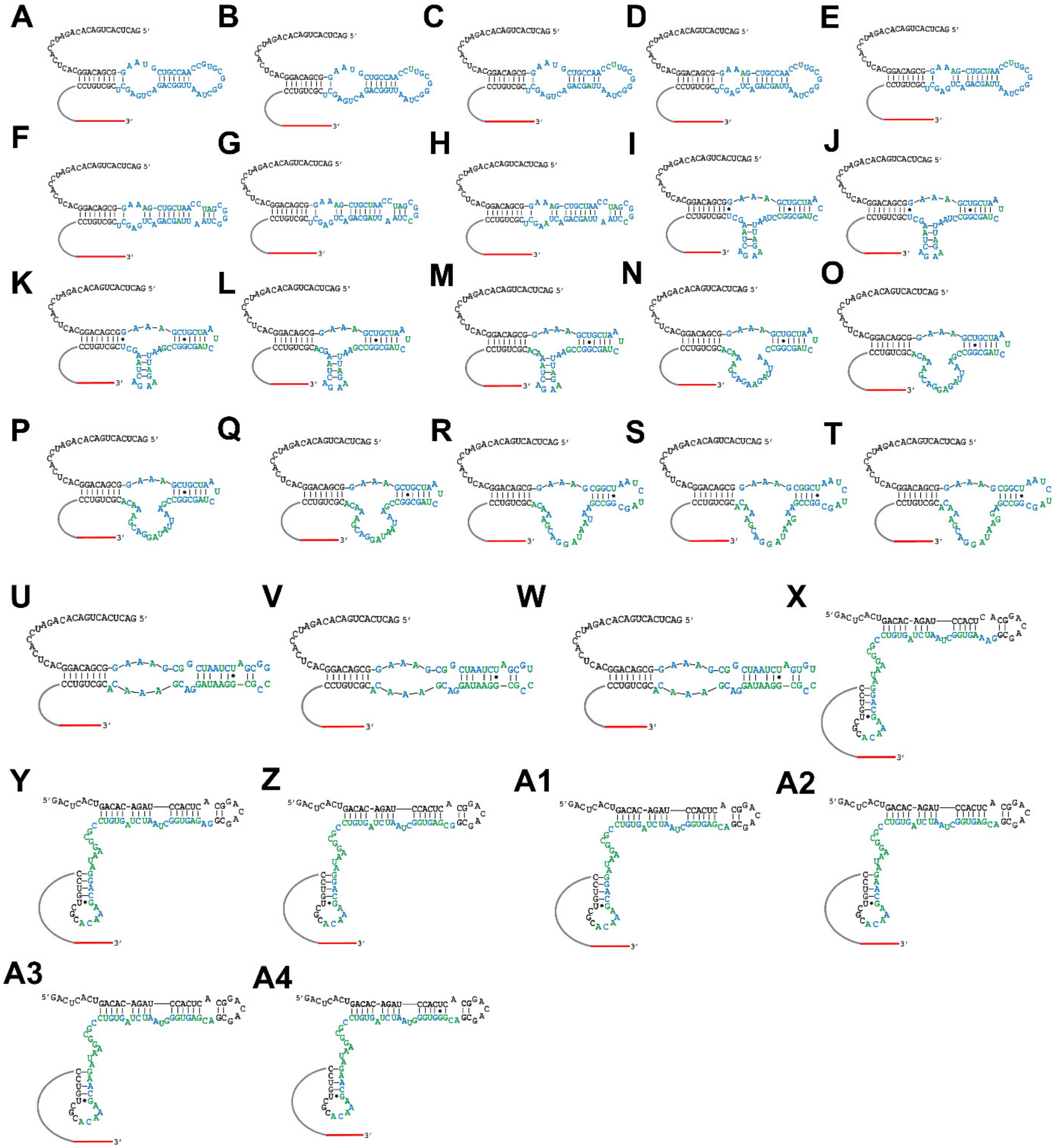

Figure S9. Computationally predicted secondary structures of active ligase intermediates Int1-Int28 that connect RS1 and CS3 via a single-step mutational pathway. AIP-ligase, RS1 (A) and PPP-ligase, CS3 (A4) are connected via mutational intermediates Int1-Int28 (B-A3) that collectively constitute a quasi-neutral pathway (see Figure 6, Table S4), enabling smooth interconversion between the two. The U_6 linker and 3′ primer sequences are depicted as gray and red lines. Invariant nucleotides are shown in black. The partially randomized region of RS1 is shown in blue in (A). Mutations to RS1 are shown in green. The 28 intermediate sequences can

be approximately classified into 7 structural folds: A-E (RS1-like), F-H, I-M, N-Q, R-T, U-W, and X-A4 (CS3-like) (see Fig. 6B). Structural changes are gradual before Int23 (X), with a notable absence of base-pairing between nucleotides from the constant (shown in black) and variable (shown in blue and green) regions. The CS3-like secondary structure emerges suddenly as a result of a single mutation to Int22 (W) and is significantly different from it. In contrast to its precursors, the CS3-like structure of Int23-Int28 (X-A3) is created by extensive base-pairing between nucleotides from the constant and variable regions.

4. REFERENCES

1. C. Kao, M. Zheng, S. Rudisser, A simple and efficient method to reduce nontemplated nucleotide addition at the 3 terminus of RNAs transcribed by T7 RNA polymerase. *RNA* **5**, 1268-1272 (1999).
2. T. Walton, S. DasGupta, D. Duzdevich, S. S. Oh, J. W. Szostak, In vitro selection of ribozyme ligases that use prebiotically plausible 2-aminoimidazole-activated substrates. *Proc Natl Acad Sci U S A* **117**, 5741-5748 (2020).
3. R. Schmieder, R. Edwards, Quality control and preprocessing of metagenomic datasets. *Bioinformatics* **27**, 863-864 (2011).
4. F. Madeira *et al.*, The EMBL-EBI search and sequence analysis tools APIs in 2019. *Nucleic Acids Res* **47**, W636-W641 (2019).
5. F. Sievers, D. G. Higgins, The Clustal Omega Multiple Alignment Package. *Methods Mol Biol* **2231**, 3-16 (2021).
6. D. H. Mathews *et al.*, Incorporating chemical modification constraints into a dynamic programming algorithm for prediction of RNA secondary structure. *Proc Natl Acad Sci U S A* **101**, 7287-7292 (2004).
7. K. N. Dyer *et al.*, High-throughput SAXS for the characterization of biomolecules in solution: a practical approach. *Methods Mol Biol* **1091**, 245-258 (2014).
8. D. J. Rosenberg, G. L. Hura, M. Hammel, Size exclusion chromatography coupled small angle X-ray scattering with tandem multiangle light scattering at the SIBYLS beamline. *Methods Enzymol* **677**, 191-219 (2022).
9. S. Classen *et al.*, Implementation and performance of SIBYLS: a dual endstation small-angle X-ray scattering and macromolecular crystallography beamline at the Advanced Light Source. *J Appl Crystallogr* **46**, 1-13 (2013).
10. J. B. Hopkins, R. E. Gillilan, S. Skou, BioXTAS RAW: improvements to a free open-source program for small-angle X-ray scattering data reduction and analysis. *J Appl Crystallogr* **50**, 1545-1553 (2017).
11. R. P. Rambo, J. A. Tainer, Accurate assessment of mass, models and resolution by small-angle scattering. *Nature* **496**, 477-481 (2013).
12. H. Liu, P. H. Zwart, Determining pair distance distribution function from SAXS data using parametric functionals. *J Struct Biol* **180**, 226-234 (2012).
13. T. D. Grant, Ab initio electron density determination directly from solution scattering data. *Nat Methods* **15**, 191-193 (2018).
14. D. Schneidman-Duhovny, M. Hammel, J. A. Tainer, A. Sali, Accurate SAXS profile computation and its assessment by contrast variation experiments. *Biophys J* **105**, 962-974 (2013).
15. E. C. Meng *et al.*, UCSF ChimeraX: Tools for structure building and analysis. *Protein Sci* **32**, e4792 (2023).
16. A. M. Watkins, R. Rangan, R. Das, FARFAR2: Improved De Novo Rosetta Prediction of Complex Global RNA Folds. *Structure* **28**, 963-976 e966 (2020).
17. W. Humphrey, A. Dalke, K. Schulten, VMD: visual molecular dynamics. *J Mol Graph* **14**, 33-38, 27-38 (1996).
18. J. C. Phillips *et al.*, Scalable molecular dynamics with NAMD. *J Comput Chem* **26**, 1781-1802 (2005).
19. J. Huang, A. D. MacKerell, Jr., CHARMM36 all-atom additive protein force field: validation based on comparison to NMR data. *J Comput Chem* **34**, 2135-2145 (2013).
